# Power-Modified Kies-Exponential Distribution: Properties, Classical and Bayesian Inference with an Application to Engineering Data

**DOI:** 10.3390/e24070883

**Published:** 2022-06-27

**Authors:** Ahmed Z. Afify, Ahmed M. Gemeay, Nada M. Alfaer, Gauss M. Cordeiro, Eslam H. Hafez

**Affiliations:** 1 Department of Statistics, Mathematics and Insurance, Benha University, Benha 13511, Egypt; 2 Department of Mathematics, Faculty of Science, Tanta University, Tanta 31527, Egypt; ahmed.gemeay@science.tanta.edu.eg; 3 Department of Mathematics & Statistics, College of Science, Taif University, P.O. Box 11099, Taif 21944, Saudi Arabia; nmfaer@tu.edu.sa; 4 Departamento de Estatística, Universidade Federal de Pernambuco, Recife 50710-165, Brazil; gauss@de.ufpe.br; 5 Mathematics Department, Faculty of Science, Helwan University, Helwan 11795, Egypt; eslamhussam@science.helwan.edu.eg

**Keywords:** Anderson–Darling estimation, Cramér–von Mises estimation, exponential distribution, mean residual life, percentile estimation, power transformation, risk measures

## Abstract

We introduce here a new distribution called the power-modified Kies-exponential (PMKE) distribution and derive some of its mathematical properties. Its hazard function can be bathtub-shaped, increasing, or decreasing. Its parameters are estimated by seven classical methods. Further, Bayesian estimation, under square error, general entropy, and Linex loss functions are adopted to estimate the parameters. Simulation results are provided to investigate the behavior of these estimators. The estimation methods are sorted, based on partial and overall ranks, to determine the best estimation approach for the model parameters. The proposed distribution can be used to model a real-life turbocharger dataset, as compared with 24 extensions of the exponential distribution.

## 1. Introduction

Exponential distribution is analytically tractable and, due to its lack of memory, is considered one of the important classical distributions. However, due to its constant hazard rate and unimodal density function, it has limited applications and cannot be adopted to a model phenomenon showing decreasing, increasing, or bathtub-shaped hazard rates. Hence, the statistical literature contains several extensions of the exponential distribution to increase its applicability and flexibility. One example is the modified Kies-exponential (MKE) introduced by Al-Babtain et al. [1] with the cumulative distribution function (CDF) and probability density function (PDF) (for t>0)

(1) $$\begin{array}{cc} G(t) & =1-\exp\left\{-{\left[\exp\left(\lambda \;t\right)-1\right]}^{\alpha }\right\},\;\;\;\;\alpha ,\lambda >0,\\ g(t) & =\alpha \;\lambda \;\exp\left(\lambda \;\alpha \;t\right){\left[1-\exp\left(-\lambda \;t\right)\right]}^{\alpha -1}\exp\left\{-{\left[\exp\left(\lambda \;t\right)-1\right]}^{\alpha }\right\}, \end{array}$$

where α is a shape parameter, and λ is a scale parameter.

The hazard rate function (HRF) of the MKE distribution can be decreasing, increasing, or bathtub-shaped. Interestingly, the two-parameter MKE model has a bathtub-shaped hazard function, whereas most distributions with this bathtub shape have problems related to algebraic complexity, an increasing number of parameters, and/or estimation problems. Hence, it can be adopted to a model phenomenon showing decreasing, increasing, or bathtub-shaped hazard rates and thus becomes more flexible than the exponential distribution for analyzing real-life data.

We propose a flexible extension of the MKE distribution named the power-modified Kies-exponential (PMKE) distribution, which provides more accuracy and flexibility for fitting real-life data. This distribution is generated based on the power transformation (P-T). The P-T has attracted attention over the years for its mathematical properties, which sometimes lead to surprising physical consequences, and for its appearance in a diverse range of natural and man-made phenomena. In fact, the generated power distributions have applications to a broad variety of different branches of human endeavor, including physics, earth sciences, biology, ecology, paleontology, computer and information sciences, engineering, and the social sciences.

The P-T of two random variables *X* and *T*, say, $X=T^{\frac{1}{\beta }}$, has been used for generating power distributions. Examples include power Lindley [2], power half-logistic [3], power Lomax [4], inverse-power Lindley [5], power binomial-exponential [6], power log-Dagum [7], power length-biased Suja [8], inverse-power logistic-exponential [9], and inverse-power Burr–Hatke [10].

The PMKE distribution has a very flexible density that can be symmetric, negatively skewed, positively skewed, or reverse-J-shaped, and it can allow for greater flexibility in the tails. It is also capable of modeling monotonically increasing, monotonically decreasing, and bathtub-shaped hazard rates. Furthermore, the CDF of this distribution has a closed form expression, which makes it ideal for applications in various fields such as engineering, reliability, life testing, survival analysis, and biomedical studies. A real data application from engineering science shows that the PMKE distribution is very competitive, with 19 extensions of the exponential distribution, including beta exponential (BE) [11], Marshall-Olkin logistic-exponential (MOLE) [12], exponentiated-exponential (ExE) [13], Harris extended-exponential (HEE) [14], Marshall-Olkin exponential (MOE) [15], and inverse-Pareto exponential distributions.

The rest of this paper is organized as follows. In Section 2, we introduce the PMKE distribution. In Section 3, we derive some of its mathematical properties. Actuarial measures of the new distribution are discussed in Section 4. Some classical methods of estimation along with detailed simulation results are reported in Section 5. Section 6 is devoted to Bayesian estimation of the parameters under different loss functions. A real-life data application is presented in Section 7. Finally, some conclusions and major findings are addressed in Section 8.

## 2. The PMKE Distribution

By applying the PT transform $X=T^{\frac{1}{\beta }}$ to (1), we obtain the CDF of the PMKE distribution (for x>0):

(2) $$\begin{array}{cc} F(x) & =1-\exp\left\{-{\left[\exp\left(\lambda \;x^{\beta }\right)-1\right]}^{\alpha }\right\},\;\;\;\alpha ,\lambda ,\beta >0, \end{array}$$

where α and β are shape parameters, and λ is a scale parameter.

Henceforth, we denote by X∼PMKE(α,λ,β) a random variable with CDF (2). The PDF and HRF of *X* are

(3) $$\begin{array}{cc} f(x) & =\alpha \;\lambda \;\beta \;x^{\beta -1}\;\exp\left(\lambda \;\alpha \;x^{\beta }\right){\left[1-\exp\left(-\lambda \;x^{\beta }\right)\right]}^{\alpha -1}\exp\left\{-{\left[\exp\left(\lambda \;x^{\beta }\right)-1\right]}^{\alpha }\right\} \end{array}$$

and

$$\begin{array}{cc} h(x) & =\alpha \;\lambda \;\beta \;x^{\beta -1}\;\exp\left(\lambda \;x^{\beta }\right)\;{\left[1-\exp\left(-\lambda \;x^{\beta }\right)\right]}^{\alpha -1}, \end{array}$$

respectively.

**Figure 1:**
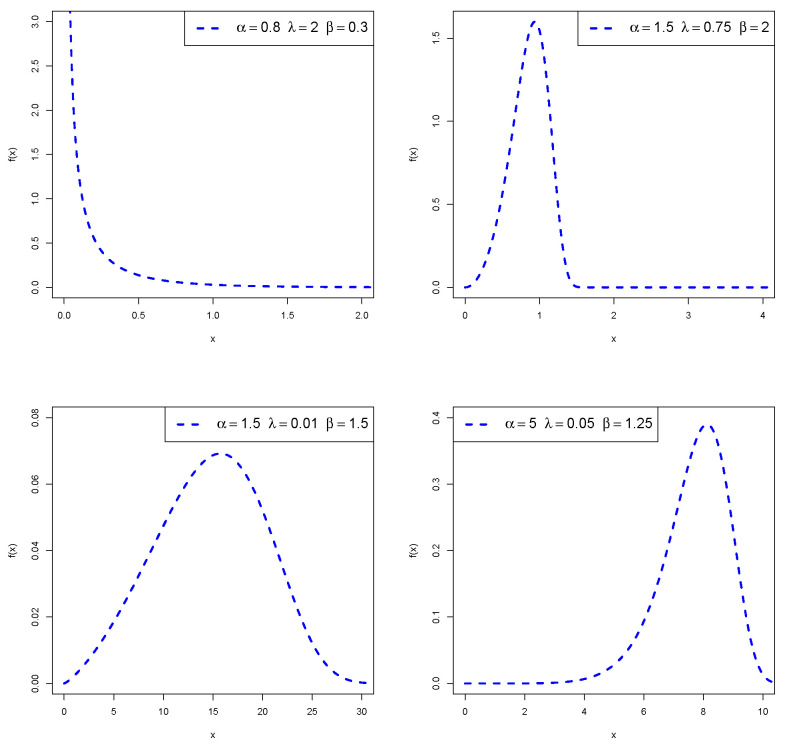
Density shapes of the PMKE distribution.

**Figure 2:**
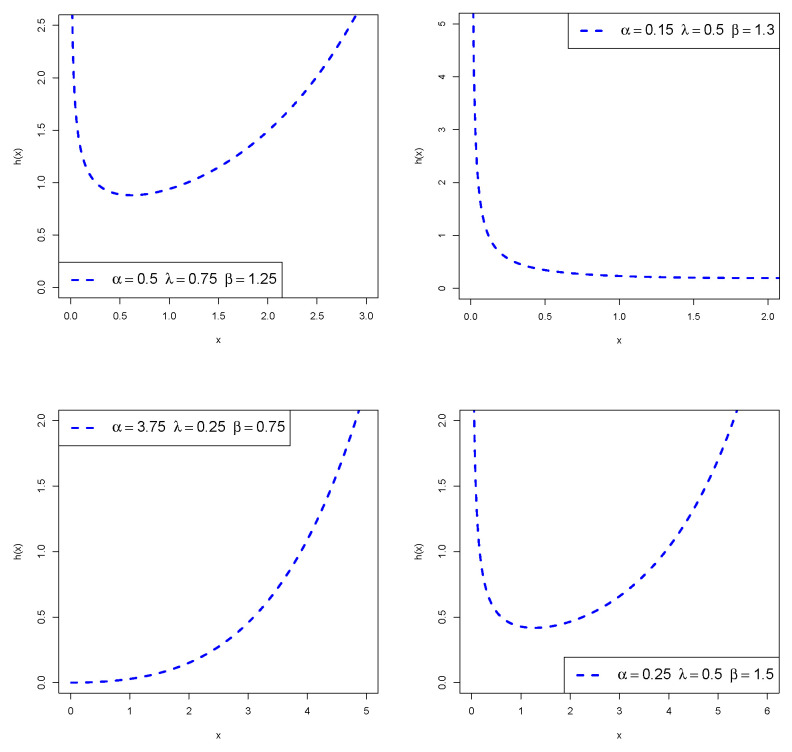
Hazard shapes of the PMKE distribution.

Plots of the PDF and HRF of *X* are displayed in Figure 1 and Figure 2, respectively. These plots reveal that the density of *X* can be left-skewed, reverse-J-shaped, or right-skewed, and its HRF can be bathtub-shaped, increasing, or decreasing.

## 3. Mathematical Properties

### 3.1. Quantile Function

The quantile function (QF) of *X* follows by inverting the CDF (2) as

(4) $$Q\left(p\right)={\left\{\frac{\log\left[{\left(-\log\left(1-p\right)\right)}^{1/\alpha }+1\right]}{\lambda }\right\}}^{1/\beta },\;0<p<1.$$

### 3.2. Linear Representation

An expansion for Equation (2) can be expressed as

$$\begin{array}{cc} F(x) & =1-\sum_{k=0}^{\infty }\frac{{\left(-1\right)}^{k}}{k!}\;{\left[\exp\left(\lambda x^{\beta }\right)-1\right]}^{k\alpha }\\ \; & =1-\sum_{k=0}^{\infty }\frac{{\left(-1\right)}^{k}}{k!}\exp\left(k\lambda \alpha x^{\beta }\right){\left[1-\exp\left(-\lambda x^{\beta }\right)\right]}^{k\alpha }\\ \; & =1-\sum_{k=0}^{\infty }\sum_{m=0}^{\infty }\frac{{\left(-1\right)}^{k+m}{\left(k\alpha \right)}_{m}}{k!m!}\exp\left[-\lambda \left(m-k\alpha \right)x^{\beta }\right], \end{array}$$

where ${\left(k\alpha \right)}_{m}=\left(k\alpha \right)\left(k\alpha -1\right)\ldots \left(k\alpha -m+1\right)$. By differentiating the last equation, we have

$$\begin{array}{c} f\left(x\right)=\sum_{k,m=0}^{\infty }\phi_{k,m}\;g_{\lambda (m-k\alpha )\beta }\left(x\right), \end{array}$$

where $g_{\lambda (m-k\alpha )\beta }\left(x\right)$ is the Weibull density with scale parameter λ(m−kα), shape parameter β, and $\phi_{k,m}={\left(-1\right)}^{k+m+1}{\left(k\alpha \right)}_{m}/\left(k!\;m!\right)$.

### 3.3. Moments

The *r*th ordinary moment of *X* can be expressed in terms of the complete gamma function

$$\begin{array}{c} \mu_{r}^{\prime }=E\left(X^{r}\right)=\int_{0}^{\infty }x^{r}\;f\left(x\right)dx=\sum_{k,m=0}^{\infty }\phi_{k,m}\;{\left[\lambda (m-k\alpha )\right]}^{-\frac{r}{\beta }}\;\Gamma \left(\frac{\beta +r}{\beta }\right). \end{array}$$

We obtain the first four ordinary moments of *X* by setting r=1,2,3, and 4. The central moments and cumulants of *X* are easily obtained from these ordinary moments.

The *s*th incomplete moment of *X* takes the form

$$\begin{array}{c} \alpha_{s}\left(t\right)=\int_{0}^{t}x^{s}\;f\left(x\right)dx=\sum_{k,m=0}^{\infty }\phi_{k,m}\;{\left[\lambda (m-k\alpha )\right]}^{-\frac{s}{\beta }}\;\gamma \left(\frac{\beta +s}{\beta },\lambda \left(m-k\alpha \right)t^{\beta }\right), \end{array}$$

where $\gamma \left(\frac{\beta +s}{\beta },\lambda \left(m-k\alpha \right)t^{\beta }\right)$ denotes the lower incomplete gamma function.

The important application of the first incomplete moment is related to the Bonferroni and Lorenz curves defined by $L\left(p\right)=\alpha_{1}\left(x_{p}\right)/\mu_{1}^{\prime }$ and $B\left(p\right)=\alpha_{1}\left(x_{p}\right)/\left(p\mu_{1}^{\prime }\right)$, respectively, where $x_{p}=Q\left(p\right)$ can be evaluated numerically by Equation (4) for a given probability *p*. These curves are very useful in economics, demography, insurance, engineering, and medicine. Another application of the first incomplete moment refers to the mean residual life (MRL) and the mean waiting time given by $m_{1}\left(t\right)=\left[1-\alpha_{1}\left(t\right)\right]/S\left(t\right)-t$ and $M_{1}\left(t\right)=t-\alpha_{1}\left(t\right)/F\left(t\right)$, respectively.

## 4. Actuarial Measures

We discuss the theoretical and computational aspects of some important risk measures, which play a crucial role in portfolio optimization under uncertainty.

The VaR of a random variable is the *q*th quantile of its CDF given by $VaR_{q}=Q\left(q\right)$ (see Artzner [16]). Therefore, the VaR of *X* can be obtained from (4).

The TVaR is used to quantify the expected value of the loss given that an event outside a given probability level has occurred. The TVaR of *X* is given by

$${\mathrm{TVaR}}_{q}=\frac{1}{\left(1-q\right)}\int_{{\mathrm{VaR}}_{q}}^{\infty }x\;\;f\left(x\right)dx,$$

which follows as

$$\begin{array}{ccc} {\mathrm{TVaR}}_{q} & = & \frac{1}{1-q}\sum_{k,m=0}^{\infty }\phi_{k,m}\int_{{\mathrm{VaR}}_{q}}^{\infty }xg_{\lambda (m-\alpha k)\beta }\left(x\right)\\ & = & \frac{1}{1-q}\sum_{k,m=0}^{\infty }\phi_{k,m}\left[\lambda (m-k\alpha )\right]\;{\mathrm{VaR}}_{q}^{\beta +1}E_{-\frac{1}{\beta }}\left[\lambda \left(m-\alpha k\right){\mathrm{VaR}}_{q}^{\beta }\right], \end{array}$$

where $E_{p}\left(z\right)=\int_{1}^{\infty }t^{-p}\;\exp\left(-tz\right)dt$ is the exponential integral.

The expected shortfall (ES) is a risk measure sensitive to the shape of the tail of the distribution of returns on a portfolio, namely,

$${\mathrm{ES}}_{q}\left(x\right)=\frac{1}{q}\int_{0}^{q}VaR_{t}\;dt.$$

Some numerical values of VaR, TVaR, and ES for four distributions are reported in Table 1. The values of these measures are obtained for four distributions at the same parameter values to investigate the tails of these models. The values of VaR, TVaR, and ES for the PMKE distribution are greater than those for the MKE distribution and the other two models, thus showing that the proposed distribution has a heavier tail than its competing models. Hence, the additional parameter β provides greater flexibility for the PMKE distribution over the MKE model.

## 5. Methods of Estimation

In this section, we discuss seven methods to estimate the parameters $\theta ={\left(\alpha ,\lambda ,\beta \right)}^{⊤}$ of the PMKE distribution and compare them by means of Monte Carlo simulations.

The AdequacyModel package for the R statistical computing environment provides a comprehensive and efficient general meta-heuristic optimization method for maximizing or minimizing an arbitrary objective function, which can be used to find the estimates of θ in the following methods. The data is accessed on 9 May 2021 and its details are available at https://rdrr.io/cran/AdequacyModel/.

### 5.1. Methods

Let $x_{1},\ldots ,x_{n}$ be a random sample of size *n* from the PDF (3). The log-likelihood function for θ reduces to

(5) $$\begin{array}{ccc} \ell & = & -\sum_{i=1}^{n}\left[\exp\left(\lambda x_{i}^{\beta }\right)-1\right]{}^{\alpha }+\left(\alpha -1\right)\sum_{i=1}^{n}\log\left[{\left(e-1\right)}^{˘x_{i}^{\mathrm{fi}}}\right]+\lambda \sum_{i=1}^{n}x_{i}^{\beta }\\ & + & \left(\beta -1\right)\sum_{i=1}^{n}\log\left(x_{i}\right)+n\log\left(\alpha \beta \lambda \right). \end{array}$$

The maximum likelihood estimate (MLE) of θ can be obtained by maximizing *ℓ*.

Let $x_{1:n},\ldots ,x_{n:n}$ be the corresponding order statistics. The ordinary least-squares estimates (OLSEs) of the parameters are determined by minimizing the function

$$O=\sum_{i=1}^{n}{\left[F\left(x_{i:n}\right)-\frac{i}{n+1}\right]}^{2}=\sum_{i=1}^{n}{\left\{1-\exp\left[-{\left(\exp\left(\lambda x_{i:n}^{\beta }\right)-1\right)}^{\alpha }\right]-\frac{i}{n+1}\right\}}^{2}.$$

Alternatively, the weighted least-squares estimators (WLSEs) can be calculated by minimizing

$$W=\sum_{i=1}^{n}\frac{{\left(n+1\right)}^{2}\left(n+2\right)}{i(n-i+1)}{\left\{1-\exp\left(-{\left[\exp\left(\lambda x_{i:n}^{\beta }\right)-1\right]}^{\alpha }\right)-\frac{i}{n+1}\right\}}^{2}.$$

Further, the Anderson–Darling estimates (ADEs) are obtained by minimizing the function

$$A=-n-\frac{1}{n}\sum_{i=1}^{n}\left(2i-1\right)\left[\log F\left(x_{i:n}\right)+\log S\left(x_{i:n}\right)\right],$$

whereas the Cramér–von Mises estimates (CVMEs) are determined by minimizing

$$CV=\frac{1}{12n}+\sum_{i=1}^{n}{\left[F\left(x_{i:n}\right)-\frac{2i-1}{2n}\right]}^{2}=\frac{1}{12n}+\sum_{i=1}^{n}{\left\{1-\exp\left[-{\left(\exp\left(\lambda x_{i:n}^{\beta }\right)-1\right)}^{\alpha }\right]-\frac{2i-1}{2n}\right\}}^{2}.$$

The maximum product of spacing estimates (MPSEs) are based on the uniform spacing

$$D_{i}=F\left(x_{i}\right)-F\left(x_{i-1}\right),$$

where $F(x_{0})=0$, $F(x_{n+1}=1)$, $\sum_{i=1}^{n+1}D_{i}=1$, and they follow by maximizing

$$G=\frac{1}{n+1}\sum_{i=1}^{n+1}\log\left(D_{i}\right).$$

Finally, the percentile estimates (PCEs) follow by minimizing

$$PCE=\sum_{i=1}^{n}{\left[x_{i:n}-Q\left(p_{i}\right)\right]}^{2}=\sum_{i=1}^{n}{\left\{x_{i:n}-{\left[\frac{\log\left({\left(-\log\left(1-p_{i}\right)\right)}^{1/\alpha }+1\right)}{\lambda }\right]}^{1/\beta }\right\}}^{2},$$

where $p_{i}=i/\left(n+1\right)$ is an estimate of $F(x_{i:n})$.

### 5.2. Monte Carlo Simulations

We explore here the performance of the aforementioned estimation methods in estimating the PMKE parameters using simulation results. We use the sample sizes n={20,50,100,200,350} and some parameter values. We generate N=1000 random samples from the PMKE distribution and calculate the average absolute biases (BIAS), mean square errors (MSEs), and mean relative estimates (MREs) using R software.

The BIAS, MSEs, and MREs have the forms

$$BIAS=\frac{1}{N}\sum_{i=1}^{N}|\hat{\theta_{i}}-\theta_{i}|,\;\;\;\;MSEs=\frac{1}{N}\sum_{i=1}^{N}{\left(\hat{\theta_{i}}-\theta_{i}\right)}^{2},\;\;\;\;MREs=\frac{1}{N}\sum_{i=1}^{N}\left|\hat{\theta_{i}}-\theta_{i}\right|/\theta_{i},$$

where $\theta_{i}$ can represent α,λ, and β.

Table 2, Table 3, Table 4, Table 5 and Table 6 report the simulation results, including the BIAS, MSEs, and MREs from the seven estimation methods. We can note that they show small BIAS, MSEs, and MREs for all parameter combinations. All seven estimators have the consistency property, where these quantities decrease when the sample size increases for all scenarios. Further, we conclude that the MLEs, ADEs, CVMEs, LSEs, MPSs, PCEs, and WLSEs are close to the true PMKE parameters.

The simulation results in Table 2, Table 3, Table 4, Table 5 and Table 6 show the ranks of the estimates among all approaches by the superscripts in each row, and the partial sum of the ranks by ∑Ranks. The partial and overall ranks of these estimates reported in Table 7 indicate the performance ordering of all estimators. According to Table 7, the performance ordering of all methods is MPSEs, MLEs, ADEs, WLSEs, PCEs, CVMEs, and LSEs. In summary, the MPSEs outperform all estimates from the other approaches for the PMKE distribution with an overall score of 38. Furthermore, the maximum likelihood can be considered a rival approach for the MPS method with an overall score of 69.5.

## 6. Bayes Estimation Method and Simulations

We obtain here the Bayes estimators of the parameters of the PMKE distribution using the symmetric and asymmetric loss functions. We have to choose a prior density function that covers our belief about the data and choose appropriate hyper-parameter values. Based on a complete sample, we adopt the square error (SE), general entropy (GE), and linear exponential (Linex) loss functions to obtain the estimates and consider that α,λ, and β are independent. We choose gamma-independent priors for the parameters, namely,

$$\pi_{1}\left(\alpha \right)\propto \alpha^{\mu_{1}-1}\exp\left(-\alpha \lambda_{1}\right),\;\;\;\;\alpha >0,\;\mu_{1},\lambda_{1}>0,$$

$$\pi_{2}\left(\beta \right)\propto \beta^{\mu_{2}-1}\exp\left(-\beta \lambda_{2}\right),\;\;\;\;\beta >0,\;\mu_{2},\lambda_{2}>0,$$

and

$$\pi_{3}\left(\lambda \right)\propto \lambda^{\mu_{3}-1}\exp\left(-\lambda \lambda_{3}\right),\;\;\;\;\lambda >0,\;\mu_{3},\lambda_{3}>0.$$

The gamma prior encourages researchers to feel confident in the data. If we do not have any belief about the data, we must adopt non-informative priors by setting the following values, so that $\mu_{i}$ tends to zero and $\lambda_{i}$ tends to infinity (i=1,2,3). In this way, we can change informative priors into non-informative priors. After this, we can find the form of the joint PDF prior of α,λ, and β as

$$\pi \left(\alpha ,\lambda ,\beta \right)\propto \alpha^{\mu_{1}-1}\beta^{\mu_{2}-1}\lambda^{\mu_{3}-1}\;\exp\left[-\left(\alpha \lambda_{1}+\beta \lambda_{2}+\lambda \lambda_{3}\right)\right],\;\;\;\;\alpha ,\beta >0.$$

Thus,

$$L\left(\alpha ,\lambda ,\beta \right)={\left(\alpha \beta \lambda \right)}^{n}\;\exp\left[\lambda \sum_{i=1}^{n}x_{i}^{\beta }-\sum_{i=1}^{n}\left(\exp\left(\lambda x_{i}^{\beta }\right)-1\right){}^{\alpha }\right]\;\prod_{i=1}^{n}x_{i}^{\beta -1}\left[\exp\left(\lambda x_{i}^{\beta }\right)-1\right]{}^{\alpha -1}.$$

By multiplying the last two equations, we obtain

(6) $$\begin{array}{ccc} \pi^{*}\left(\alpha ,\lambda ,\beta \right) & \propto & L\left(\alpha ,\lambda ,\beta \right)\pi \left(\alpha ,\lambda ,\beta \right)\propto \alpha^{\mu_{1}-1+n}\beta^{\mu_{2}-1+n}\lambda^{\mu_{3}-1+n}\times \\ & & \exp\left\{\left[\lambda \sum_{i=1}^{n}x_{i}^{\beta }-\sum_{i=1}^{n}\left(\exp\left(\lambda x_{i}^{\beta }\right)-1\right){}^{\alpha }\right]-(\alpha \lambda_{1}+\beta \lambda_{2}+\lambda \lambda_{3})\right\}. \end{array}$$

According to the SE loss function, the Bayes estimator of B=B(θ), where θ=(α,λ,β), is

(7) $${\hat{B}}_{SE}=\int_{\theta }B\;\pi^{*}\left(\theta \right)\;d\theta ,$$

where $\pi^{*}\left(\theta \right)$ is given by Equation (6). The Bayes estimator under the LINEX loss function is the value of

(8) $$\begin{array}{c} {\hat{B}}_{\mathrm{Linex}}=\frac{-1}{c}\;\log\left(E_{\theta }\left[\exp\left(-c\theta \right)\right]\right), \end{array}$$

such that $E_{\theta }\left[\exp\left(-c\theta \right)\right]$ exists.

The Bayes estimate ${\hat{\Theta }}_{GE}$ under the GE loss function is

(9) $$\begin{array}{c} {\hat{B}}_{\mathrm{GE}}={\left(E_{\theta }\left[\theta^{-q}\right]\right)}^{\frac{-1}{q}} \end{array}$$

such that $E_{\theta }\left[\theta^{-q}\right]$ exists.

We cannot find a result for the integrals in Equations (7)–(9). Thus, we use the Markov Chain Monte Carlo (MCMC) technique to approximate these integrals and consider the Metropolis–Hastings algorithm as an example of the MCMC technique to find the estimates.

### 6.1. The MCMC Method

We adopt the MCMC method here because we do not have a well-known distribution for the posterior density function. We then calculate the BEs of α,λ, and β under the conditional posterior distribution functions for these parameters:

$$\begin{array}{cc} \pi^{*}\left(\alpha |\beta ,\lambda \right) & \propto \alpha^{\mu_{1}-1+n}\times \exp\left\{\left[\lambda \sum_{i=1}^{n}x_{i}^{\beta }-\sum_{i=1}^{n}\left(\exp\left(\lambda x_{i}^{\beta }\right)-1\right){}^{\alpha }\right]-\alpha \lambda_{1}\right\}, \end{array}$$

$$\begin{array}{cc} \pi^{*}\left(\beta |\alpha ,\lambda \right) & \propto \beta^{\mu_{2}-1+n}\times \exp\left[\left(\lambda \sum_{i=1}^{n}x_{i}^{\beta }-\sum_{i=1}^{n}\left(\exp\left(\lambda x_{i}^{\beta }\right)-1\right){}^{\alpha }\right)-\beta \lambda_{2}\right], \end{array}$$

and

$$\begin{array}{cc} \pi^{*}\left(\lambda |\alpha ,\beta \right) & \propto \beta^{\mu_{3}-1+n}\times \exp\left[\left(\lambda \sum_{i=1}^{n}x_{i}^{\beta }-\sum_{i=1}^{n}\left(\exp\left(\lambda x_{i}^{\beta }\right)-1\right){}^{\alpha }\right)-\lambda \lambda_{3}\right]. \end{array}$$

Therefore, we do not have closed forms for the conditional posterior distributions for these parameters since they do not represent any known distribution. We use the Metropolis–Hasting algorithm below to explain the steps required to compute the Bayes estimates for B=B(α,λ,β) under the SE loss function.

### 6.2. The Metropolis–Hastings Algorithm

The Metropolis–Hastings algorithm can be considered as an MCMC method for generating data from any CDF. These generated samples can be used to approximate the distribution or to compute an integral (e.g., an expected value). We use the MCMC algorithm because it is sometimes difficult to obtain samples and the posterior comes from an unknown distribution.

1. The starting values are as follows: $\alpha^{\left(0\right)}={\hat{\alpha }}_{MLE},$ $\beta^{\left(0\right)}={\hat{\beta }}_{MLE}$, $\lambda^{\left(0\right)}={\hat{\lambda }}_{MLE}.$
2. Set i = 1.
3. Generate $\alpha^{\left(*\right)}$ from the proposal distribution $N(\alpha^{\left(i-1\right)},\mathrm{Var}\left(\alpha^{\left(i-1\right)}\right).$
4. Calculate the acceptance probability $r\left(\alpha^{\left(i-1\right)},\alpha^{\left(*\right)}\right)=\min\left[1,\frac{\pi (\alpha^{\left(*\right)})}{\pi (\alpha^{\left(i-1\right)})}\right].$
5. Generate U from a uniform on (0,1).
6. If $U<r(\alpha^{\left(i-1\right)},\alpha^{\left(*\right)})$ accept the proposal distribution and set $\alpha^{\left(i\right)}=\alpha^{\left(*\right)}.$ Otherwise, reject the proposal distribution and set $\alpha^{\left(i\right)}=\alpha^{\left(i-1\right)}.$
7. Set i=i+1.
8. Repeat Steps 3–9 *N* times.
9. Obtain the BEs of α using MCMC under the SEL function as ${\hat{\alpha }}_{SE}=\sum_{i=M+1}^{N}\frac{1}{N-M}\alpha^{\left(i\right)}.$
10. Obtain the BEs of α using MCMC under the LINEX function as ${\hat{\alpha }}_{Linex}=\frac{-1}{c}\log\frac{\sum_{i=M+1}^{N}\exp\left(-c\alpha^{\left(i\right)}\right)}{N-M}$.
11. Obtain the BEs of α using MCMC under the GE function ${\hat{\alpha }}_{GE}={\left[\frac{\sum_{i=M+1}^{N}{\left(\theta^{\left(i\right)}\right)}^{-q}}{N-M}\right]}^{\frac{-1}{q}}$, where M is nburn units, and N is the number of MCMC iterations.
12. Perform Steps 3–11 to find the estimates of β,λ.

Table 8 and Table 9 report the simulation results including BIAS, MSEs, and MREs from the Bayesian estimators under three loss functions. We can note that they show small BIAS, MSEs, and MREs for all parameter combinations. The Bayesian estimates under the three loss functions have the consistency property, where these quantities decrease when the sample size increases for all scenarios. Further, all estimates are close to the true PMKE parameters.

The simulated results in Table 8 and Table 9 show the ranks of the estimates under different loss functions by the superscripts in each row, and the partial sum of the ranks by ∑Ranks. The partial and overall ranks of the explored estimates are listed in Table 7, indicating the performance ordering of all estimators. According to Table 10, the Bayesian estimates’ performance ordering is BGE, BLN, and BSE.

## 7. Application

We consider here a real dataset representing the failure times $\left(10^{3}\;h\right)$ of a turbocharger of one type of engine with 40 observations.

We compare the proposed distribution with some other well-known distributions including MKE, APE, alpha power-exponentiated exponential (APExE), BE, ExE, MOE, MOLE, HEE, GOLLE, gamma-exponentiated exponential (GExE) [17], inverse-power logistic-exponential (IPLE) [9], Kumaraswamy exponential (KE), linear exponential (LNE) [18], logistic-exponential (LE) [19], Nadarajah–Haghighi exponential (NHE) [20], transmuted exponential (TE) [18], transmuted generalized exponential (TGE) [21], and exponential (E) distributions.

These models can be compared using discrimination measures such as Akaike information (AKI), consistent Akaike information (CAKI), Bayesian information (BAI), and Hannan–Quinn information (HAQUI) criteria. Further discrimination measures include Anderson Darling (ANDA), Cramér–von Mises (CRVMI), and Kolmogorov–Smirnov (KOSM) (with its *p*-value).

**Figure 3:**
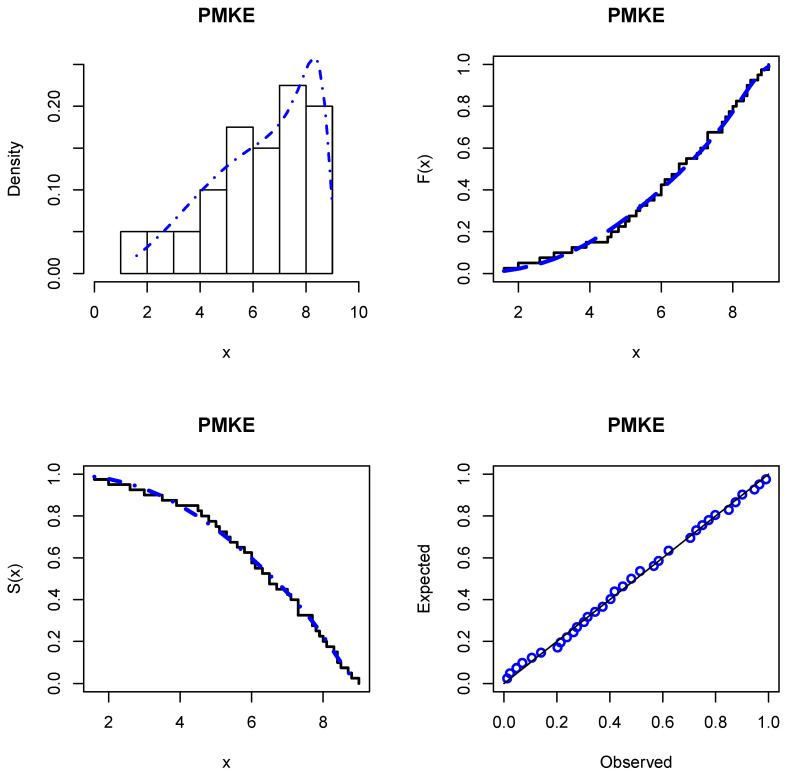
Histogram of the data with the estimated PDF, CDF, and SF of the PMKE model and P-P plot.

The MLEs and the analytical measures are calculated using the Wolfram Mathematica software (version 10). Table 11 provides analytical measures and the MLEs and their standard errors (SEs) in parentheses. The results in these tables indicate that the PMKE distribution provides a better fit than the other competing models and could be chosen as an adequate model to analyze the current data. The estimated PDF, CDF, SF, and P-P plots from the new distribution fitted to these data are reported in Figure 3.

A comparison of the PMKE distribution with its MKE sub-model using the likelihood ratio statistic (LR) is performed to check the hypotheses $H_{0}:\beta =1$ vs. $H_{1}$. The LR statistic is equal to 7.124 and its *p*-value =0.0076, which rejects $H_{0}$. Hence, the new PMKE distribution yields a superior fit to these data than the MKE distribution.

## 8. Conclusions

We have introduced a new continuous model called the power-modified Kies-exponential (PMKE) distribution and have derived some of its mathematical properties. The new density function can take different shapes. Furthermore, the PMKE failure rate function can be monotonically increasing, monotonically decreasing, or bathtub-shaped. We have also calculated some of its actuarial measures. We considered seven classical and Bayesian methods to estimate the parameters based on a complete sample. An extensive simulation study has been conducted to compare the performance of the estimates from the seven estimation methods. Based on our study, the classical maximum product of the spacing approach is recommended to estimate the PMKE parameters. The Bayesian approach provides more accurate estimates under general entropy and linear exponential loss functions than the square error loss function. A real data analysis shows that the new distribution provides a better fit than other distributions.

## Figures and Tables

**Table 1:** VaR, TVaR, and ES for four distributions.

Significance Level	PMKE	MKE	ExE	E
VaR				
	(α=0.25,λ=0.75,β=0.25)	(α=0.25,λ=0.75)	(λ=0.75,c=0.5)	λ=0.75
0.60	1.28404	0.7182	1.98496	1.21777
0.65	3.57919	1.07393	2.1847	1.39524
0.70	11.09314	1.53636	2.41038	1.60011
0.75	31.48176	2.10565	2.67215	1.84242
0.80	80.60751	2.78512	2.98681	2.13898
0.85	194.2603	3.59475	3.3858	2.52131
0.90	466.7491	4.59656	3.93951	3.06019
0.95	1255.667	5.99892	4.87237	3.98139
	(α=0.5,λ=0.1,β=0.5)	(α=0.5,λ=0.1)	(λ=0.1,c=2)	λ=0.1
0.60	38.50023	6.17476	4.48536	9.17685
0.65	57.23762	7.51664	5.5054	10.51419
0.70	83.00216	9.0558	6.73862	12.05805
0.75	118.0717	10.83025	8.2594	13.88404
0.80	165.9891	12.90384	10.19268	16.11887
0.85	233.138	15.39531	12.77318	19.00006
0.90	333.5195	18.56321	16.5283	23.06088
0.95	510.761	23.1784	23.14187	30.0029
TVaR				
	(α=0.25,λ=0.75,β=0.25)	(α=0.25,λ=0.75)	(λ=0.75,c=0.5)	λ=0.75
0.60	587.6448	3.29354	3.38959	2.54679
0.65	671.2805	3.63728	3.57624	2.72426
0.70	782.0503	4.02746	3.78978	2.92913
0.75	934.5137	4.47054	4.0402	3.17144
0.80	1154.989	4.97924	4.34423	3.468
0.85	1496.793	5.57986	4.73321	3.85034
0.90	2090.229	6.33229	5.27747	4.38921
0.95	3398.148	7.41915	6.2014	5.31041
	(α=0.5,λ=0.1,β=0.5)	(α=0.5,λ=0.1)	(λ=0.1,c=2)	λ=0.1
0.60	250.8306	14.57897	13.25437	19.19206
0.65	279.898	15.6859	14.43648	20.52941
0.70	314.9723	16.92214	15.82557	22.07326
0.75	357.9961	18.32231	17.49666	23.89946
0.80	412.3591	19.94329	19.57472	26.13408
0.85	483.9588	21.88863	22.29471	29.01554
0.90	586.1592	24.38062	26.18471	33.0764
0.95	760.5773	28.07958	32.92862	40.01812
ES				
	(α=0.25,λ=0.75,β=0.25)	(α=0.25,λ=0.75)	(λ=0.75,c=0.5)	λ=0.75
0.60	1.16312	0.11892	1.0697	0.51717
0.65	1.31938	0.17803	1.14765	0.57774
0.70	1.77238	0.2579	1.2296	0.64326
0.75	2.03646	0.36151	1.31681	0.71488
0.80	5.19745	0.49115	1.41101	0.79428
0.85	12.51398	0.64917	1.51491	0.88408
0.90	29.03652	0.83949	1.63323	0.989
0.95	68.68255	1.07138	1.77641	1.11947
	(α=0.5,λ=0.1,β=0.5)	(α=0.5,λ=0.1)	(λ=0.1,c=2)	λ=0.1
0.60	6.63995	1.85757	1.38423	3.89732
0.65	9.77218	2.2401	1.66082	4.35373
0.70	14.03504	2.67069	1.97803	4.84748
0.75	19.74208	3.15407	2.34422	5.3872
0.80	27.3055	3.69684	2.77172	5.9855
0.85	37.32049	4.30921	3.2801	6.66226
0.90	50.77811	5.00896	3.90424	7.45289
0.95	69.77617	5.83384	4.72195	8.43611

**Table 2:** Simulation results for the PMKE distribution with α=0.25, λ=0.5, and β=0.75.

*n*	Est.	Est. Par.	MLEs	ADEs	CVMEs	MPSs	LSEs	PCEs	WLSEs
20	ABBs	$\hat{\alpha }$	$0.10547^{1}$	$0.1651^{3}$	$0.20456^{6}$	$0.15261^{2}$	$0.19427^{5}$	$0.20905^{7}$	$0.18316^{4}$
		$\hat{\lambda }$	$0.23154^{2}$	$0.24381^{4}$	$0.26215^{6}$	$0.22327^{1}$	$0.25096^{5}$	$0.29056^{7}$	$0.24063^{3}$
		$\hat{\beta }$	$0.269^{1}$	$0.30396^{3}$	$0.36149^{7}$	$0.30204^{2}$	$0.35745^{6}$	$0.30548^{4}$	$0.33803^{5}$
	MSEs	$\hat{\alpha }$	$0.02658^{1}$	$0.05572^{3}$	$0.0786^{7}$	$0.04614^{2}$	$0.07008^{5}$	$0.07713^{6}$	$0.06487^{4}$
		$\hat{\lambda }$	$0.07371^{2}$	$0.07921^{4}$	$0.08998^{6}$	$0.06928^{1}$	$0.08217^{5}$	$0.11221^{7}$	$0.07723^{3}$
		$\hat{\beta }$	$0.10309^{1}$	$0.12297^{3}$	$0.15811^{7}$	$0.12032^{2}$	$0.15647^{6}$	$0.12364^{4}$	$0.14237^{5}$
	MREs	$\hat{\alpha }$	$0.42189^{1}$	$0.66042^{3}$	$0.81824^{6}$	$0.61043^{2}$	$0.77709^{5}$	$0.8362^{7}$	$0.73264^{4}$
		$\hat{\lambda }$	$0.46307^{2}$	$0.48762^{4}$	$0.52431^{6}$	$0.44655^{1}$	$0.50193^{5}$	$0.58112^{7}$	$0.48125^{3}$
		$\hat{\beta }$	$0.35867^{1}$	$0.40528^{3}$	$0.48199^{7}$	$0.40273^{2}$	$0.4766^{6}$	$0.4073^{4}$	$0.4507^{5}$
	∑Ranks		$12^{1}$	$30^{3}$	$58^{7}$	$15^{2}$	$48^{5}$	$53^{6}$	$36^{4}$
50	ABBs	$\hat{\alpha }$	$0.05994^{1}$	$0.1124^{3}$	$0.13081^{6}$	$0.08596^{2}$	$0.13006^{5}$	$0.17207^{7}$	$0.11816^{4}$
		$\hat{\lambda }$	$0.14877^{1}$	$0.18915^{4}$	$0.1966^{5}$	$0.16195^{2}$	$0.19892^{6}$	$0.2052^{7}$	$0.18569^{3}$
		$\hat{\beta }$	$0.16898^{1}$	$0.23083^{4}$	$0.27988^{6}$	$0.1916^{2}$	$0.2812^{7}$	$0.22263^{3}$	$0.24201^{5}$
	MSEs	$\hat{\alpha }$	$0.00728^{1}$	$0.03134^{3}$	$0.03862^{6}$	$0.0159^{2}$	$0.03831^{5}$	$0.05597^{7}$	$0.03171^{4}$
		$\hat{\lambda }$	$0.03537^{1}$	$0.05115^{4}$	$0.05264^{5}$	$0.03995^{2}$	$0.05384^{6}$	$0.0666^{7}$	$0.04888^{3}$
		$\hat{\beta }$	$0.04627^{1}$	$0.07953^{4}$	$0.10797^{6}$	$0.05666^{2}$	$0.10921^{7}$	$0.07077^{3}$	$0.08584^{5}$
	MREs	$\hat{\alpha }$	$0.23974^{1}$	$0.4496^{3}$	$0.52326^{6}$	$0.34385^{2}$	$0.52025^{5}$	$0.68829^{7}$	$0.47262^{4}$
		$\hat{\lambda }$	$0.29755^{1}$	$0.37831^{4}$	$0.39319^{5}$	$0.3239^{2}$	$0.39784^{6}$	$0.4104^{7}$	$0.37139^{3}$
		$\hat{\beta }$	$0.2253^{1}$	$0.30777^{4}$	$0.37317^{6}$	$0.25546^{2}$	$0.37493^{7}$	$0.29683^{3}$	$0.32269^{5}$
	∑Ranks		$9^{1}$	$33^{3}$	$51^{5.5}$	$18^{2}$	$54^{7}$	$51^{5.5}$	$36^{4}$
100	ABs	$\hat{\alpha }$	$0.04284^{1}$	$0.06749^{3}$	$0.09533^{6}$	$0.0512^{2}$	$0.09485^{5}$	$0.13417^{7}$	$0.07191^{4}$
		$\hat{\lambda }$	$0.09809^{1}$	$0.14396^{3}$	$0.1505^{6}$	$0.1118^{2}$	$0.14914^{5}$	$0.17052^{7}$	$0.14857^{4}$
		$\hat{\beta }$	$0.09306^{1}$	$0.1661^{4}$	$0.21948^{7}$	$0.11674^{2}$	$0.21413^{6}$	$0.16475^{3}$	$0.18097^{5}$
	MSEs	$\hat{\alpha }$	$0.00529^{1}$	$0.01059^{3}$	$0.02113^{6}$	$0.00548^{2}$	$0.02029^{5}$	$0.03617^{7}$	$0.01106^{4}$
		$\hat{\lambda }$	$0.01913^{1}$	$0.03043^{3}$	$0.03331^{6}$	$0.02105^{2}$	$0.03208^{4}$	$0.04839^{7}$	$0.03321^{5}$
		$\hat{\beta }$	$0.01743^{1}$	$0.04344^{4}$	$0.07185^{7}$	$0.02306^{2}$	$0.06906^{6}$	$0.04316^{3}$	$0.04985^{5}$
	MREs	$\hat{\alpha }$	$0.17137^{1}$	$0.26998^{3}$	$0.38132^{6}$	$0.20479^{2}$	$0.37941^{5}$	$0.53666^{7}$	$0.28764^{4}$
		$\hat{\lambda }$	$0.19618^{1}$	$0.28793^{3}$	$0.30099^{6}$	$0.2236^{2}$	$0.29829^{5}$	$0.34105^{7}$	$0.29713^{4}$
		$\hat{\beta }$	$0.12409^{1}$	$0.22146^{4}$	$0.29264^{7}$	$0.15566^{2}$	$0.2855^{6}$	$0.21967^{3}$	$0.24129^{5}$
	∑Ranks		$9^{1}$	$30^{3}$	$57^{7}$	$18^{2}$	$47^{5}$	$51^{6}$	$40^{4}$
200	ABBs	$\hat{\alpha }$	$0.03007^{1}$	$0.04459^{3}$	$0.0593^{5}$	$0.03615^{2}$	$0.06226^{6}$	$0.10307^{7}$	$0.04857^{4}$
		$\hat{\lambda }$	$0.06325^{1}$	$0.09736^{3}$	$0.11607^{6}$	$0.07898^{2}$	$0.12006^{7}$	$0.11247^{4}$	$0.11304^{5}$
		$\hat{\beta }$	$0.06064^{1}$	$0.11548^{3}$	$0.15909^{7}$	$0.08547^{2}$	$0.15557^{6}$	$0.11585^{4}$	$0.13411^{5}$
	MSEs	$\hat{\alpha }$	$0.00343^{2}$	$0.00408^{3}$	$0.00717^{5}$	$0.00235^{1}$	$0.00911^{6}$	$0.02137^{7}$	$0.00415^{4}$
		$\hat{\lambda }$	$0.01161^{2}$	$0.01485^{3}$	$0.02038^{5}$	$0.01118^{1}$	$0.02118^{6}$	$0.02333^{7}$	$0.01893^{4}$
		$\hat{\beta }$	$0.01027^{1}$	$0.02153^{4}$	$0.0395^{7}$	$0.01284^{2}$	$0.039^{6}$	$0.021^{3}$	$0.02795^{5}$
	MREs	$\hat{\alpha }$	$0.12029^{1}$	$0.17836^{3}$	$0.23719^{5}$	$0.14459^{2}$	$0.24904^{6}$	$0.41227^{7}$	$0.19428^{4}$
		$\hat{\lambda }$	$0.1265^{1}$	$0.19471^{3}$	$0.23214^{6}$	$0.15795^{2}$	$0.24011^{7}$	$0.22495^{4}$	$0.22607^{5}$
		$\hat{\beta }$	$0.08085^{1}$	$0.15397^{3}$	$0.21212^{7}$	$0.11395^{2}$	$0.20743^{6}$	$0.15447^{4}$	$0.17881^{5}$
	∑Ranks		$11^{1}$	$28^{3}$	$53^{6}$	$16^{2}$	$56^{7}$	$47^{5}$	$41^{4}$
350	ABBs	$\hat{\alpha }$	$0.02496^{2}$	$0.03014^{3}$	$0.04105^{5}$	$0.02376^{1}$	$0.04493^{6}$	$0.06871^{7}$	$0.03323^{4}$
		$\hat{\lambda }$	$0.04739^{1}$	$0.07593^{3}$	$0.08991^{5}$	$0.05012^{2}$	$0.0913^{6}$	$0.09237^{7}$	$0.08069^{4}$
		$\hat{\beta }$	$0.04054^{1}$	$0.0838^{3}$	$0.12006^{6}$	$0.05366^{2}$	$0.12382^{7}$	$0.08514^{4}$	$0.09207^{5}$
	MSEs	$\hat{\alpha }$	$0.00416^{6}$	$0.00168^{2}$	$0.00329^{4}$	$0.00106^{1}$	$0.00348^{5}$	$0.01005^{7}$	$0.00196^{3}$
		$\hat{\lambda }$	$0.00819^{2}$	$0.00964^{3}$	$0.01211^{5}$	$0.00475^{1}$	$0.01271^{6}$	$0.0156^{7}$	$0.0101^{4}$
		$\hat{\beta }$	$0.00588^{2}$	$0.01169^{3}$	$0.023^{6}$	$0.00567^{1}$	$0.02354^{7}$	$0.01188^{4}$	$0.01325^{5}$
	MREs	$\hat{\alpha }$	$0.09986^{2}$	$0.12057^{3}$	$0.1642^{5}$	$0.09502^{1}$	$0.17974^{6}$	$0.27486^{7}$	$0.13294^{4}$
		$\hat{\lambda }$	$0.09477^{1}$	$0.15186^{3}$	$0.17982^{5}$	$0.10023^{2}$	$0.1826^{6}$	$0.18474^{7}$	$0.16138^{4}$
		$\hat{\beta }$	$0.05406^{1}$	$0.11174^{3}$	$0.16008^{6}$	$0.07155^{2}$	$0.16509^{7}$	$0.11352^{4}$	$0.12276^{5}$
	∑Ranks		$18^{2}$	$26^{3}$	$47^{5}$	$13^{1}$	$56^{7}$	$54^{6}$	$38^{4}$

**Table 3:** Simulation results for the PMKE distribution with α=1.5, λ=0.75, and β=0.5.

*n*	Est.	Est. Par.	MLEs	ADEs	CVMEs	MPSs	LSEs	PCEs	WLSEs
20	BIAS	$\hat{\alpha }$	$0.45835^{3}$	$0.45881^{4}$	$0.46621^{7}$	$0.42896^{1}$	$0.46615^{6}$	$0.46557^{5}$	$0.45507^{2}$
		$\hat{\lambda }$	$0.07743^{7}$	$0.07404^{6}$	$0.07255^{5}$	$0.06999^{3}$	$0.07194^{4}$	$0.06983^{2}$	$0.06305^{1}$
		$\hat{\beta }$	$0.20127^{7}$	$0.166^{2}$	$0.18587^{5}$	$0.15415^{1}$	$0.17612^{4}$	$0.19208^{6}$	$0.1726^{3}$
	MSEs	$\hat{\alpha }$	$0.22203^{4}$	$0.22051^{3}$	$0.22818^{7}$	$0.20891^{1}$	$0.22806^{6}$	$0.22643^{5}$	$0.21985^{2}$
		$\hat{\lambda }$	$0.00982^{7}$	$0.00943^{6}$	$0.00913^{5}$	$0.0084^{3}$	$0.00834^{2}$	$0.00847^{4}$	$0.00718^{1}$
		$\hat{\beta }$	$0.06072^{7}$	$0.04218^{2}$	$0.05277^{6}$	$0.03402^{1}$	$0.04492^{4}$	$0.05181^{5}$	$0.04484^{3}$
	MREs	$\hat{\alpha }$	$0.30557^{3}$	$0.30587^{4}$	$0.31081^{7}$	$0.28597^{1}$	$0.31077^{6}$	$0.31038^{5}$	$0.30338^{2}$
		$\hat{\lambda }$	$0.10323^{7}$	$0.09872^{6}$	$0.09674^{5}$	$0.09331^{3}$	$0.09592^{4}$	$0.0931^{2}$	$0.08406^{1}$
		$\hat{\beta }$	$0.40254^{7}$	$0.33201^{2}$	$0.37174^{5}$	$0.3083^{1}$	$0.35223^{4}$	$0.38417^{6}$	$0.34521^{3}$
	∑Ranks		$52^{6.5}$	$35^{3}$	$52^{6.5}$	$15^{1}$	$40^{4.5}$	$40^{4.5}$	$18^{2}$
50	BIAS	$\hat{\alpha }$	$0.44961^{4}$	$0.43566^{2}$	$0.45982^{6}$	$0.39747^{1}$	$0.46208^{7}$	$0.45708^{5}$	$0.44484^{3}$
		$\hat{\lambda }$	$0.04714^{6}$	$0.04219^{3}$	$0.04778^{7}$	$0.04033^{1}$	$0.04711^{5}$	$0.04455^{4}$	$0.04088^{2}$
		$\hat{\beta }$	$0.18746^{7}$	$0.15016^{1}$	$0.17385^{4}$	$0.15108^{2}$	$0.17703^{6}$	$0.17565^{5}$	$0.15643^{3}$
	MSEs	$\hat{\alpha }$	$0.21603^{4}$	$0.20735^{2}$	$0.22272^{6}$	$0.19354^{1}$	$0.22423^{7}$	$0.22124^{5}$	$0.21319^{3}$
		$\hat{\lambda }$	$0.00412^{7}$	$0.00294^{2}$	$0.00399^{6}$	$0.00269^{1}$	$0.00366^{5}$	$0.00341^{4}$	$0.00296^{3}$
		$\hat{\beta }$	$0.04687^{7}$	$0.03029^{1}$	$0.04154^{5}$	$0.03163^{2}$	$0.04236^{6}$	$0.04078^{4}$	$0.03357^{3}$
	MREs	$\hat{\alpha }$	$0.29974^{4}$	$0.29044^{2}$	$0.30655^{6}$	$0.26498^{1}$	$0.30805^{7}$	$0.30472^{5}$	$0.29656^{3}$
		$\hat{\lambda }$	$0.06286^{6}$	$0.05625^{3}$	$0.0637^{7}$	$0.05377^{1}$	$0.06281^{5}$	$0.0594^{4}$	$0.0545^{2}$
		$\hat{\beta }$	$0.37492^{7}$	$0.30032^{1}$	$0.34771^{4}$	$0.30215^{2}$	$0.35406^{6}$	$0.3513^{5}$	$0.31287^{3}$
	∑Ranks		$52^{6}$	$17^{2}$	$51^{5}$	$12^{1}$	$54^{7}$	$41^{4}$	$25^{3}$
100	BIAS	$\hat{\alpha }$	$0.41393^{2}$	$0.42517^{4}$	$0.43929^{6}$	$0.34777^{1}$	$0.43885^{5}$	$0.44035^{7}$	$0.42266^{3}$
		$\hat{\lambda }$	$0.03267^{7}$	$0.03099^{3}$	$0.03185^{6}$	$0.03042^{2}$	$0.03161^{4}$	$0.03168^{5}$	$0.02972^{1}$
		$\hat{\beta }$	$0.15646^{5}$	$0.14163^{2}$	$0.16716^{7}$	$0.12756^{1}$	$0.15626^{4}$	$0.16294^{6}$	$0.15093^{3}$
	MSEs	$\hat{\alpha }$	$0.1921^{2}$	$0.20049^{4}$	$0.20996^{6}$	$0.168^{1}$	$0.20882^{5}$	$0.21015^{7}$	$0.19852^{3}$
		$\hat{\lambda }$	$0.00178^{7}$	$0.00168^{3}$	$0.00169^{4}$	$0.00155^{1}$	$0.00176^{6}$	$0.0017^{5}$	$0.0016^{2}$
		$\hat{\beta }$	$0.03311^{5}$	$0.02993^{2}$	$0.03658^{7}$	$0.0229^{1}$	$0.03212^{4}$	$0.03352^{6}$	$0.03011^{3}$
	MREs	$\hat{\alpha }$	$0.27595^{2}$	$0.28344^{4}$	$0.29286^{6}$	$0.23184^{1}$	$0.29257^{5}$	$0.29357^{7}$	$0.28177^{3}$
		$\hat{\lambda }$	$0.04356^{7}$	$0.04132^{3}$	$0.04247^{6}$	$0.04057^{2}$	$0.04215^{4}$	$0.04224^{5}$	$0.03962^{1}$
		$\hat{\beta }$	$0.31292^{5}$	$0.29327^{2}$	$0.33432^{7}$	$0.25512^{1}$	$0.31253^{4}$	$0.32588^{6}$	$0.30185^{3}$
	∑Ranks		$42^{5}$	$27^{3}$	$55^{7}$	$11^{1}$	$41^{4}$	$54^{6}$	$22^{2}$
200	BIAS	$\hat{\alpha }$	$0.38589^{3}$	$0.38271^{2}$	$0.4126^{6}$	$0.31596^{1}$	$0.41093^{5}$	$0.415^{7}$	$0.3901^{4}$
		$\hat{\lambda }$	$0.0247^{6}$	$0.02191^{1}$	$0.02271^{4}$	$0.02277^{5}$	$0.02485^{7}$	$0.02262^{3}$	$0.02209^{2}$
		$\hat{\beta }$	$0.14498^{6}$	$0.13511^{2}$	$0.14291^{5}$	$0.11632^{1}$	$0.15041^{7}$	$0.14008^{4}$	$0.13984^{3}$
	MSEs	$\hat{\alpha }$	$0.17273^{3}$	$0.17228^{2}$	$0.19221^{6}$	$0.15037^{1}$	$0.19112^{5}$	$0.19246^{7}$	$0.17676^{4}$
		$\hat{\lambda }$	$0.00107^{7}$	$0.00077^{1}$	$0.00095^{5}$	$0.00092^{4}$	$0.00104^{6}$	$0.00084^{2}$	$0.00085^{3}$
		$\hat{\beta }$	$0.02771^{6}$	$0.02422^{2}$	$0.02688^{5}$	$0.02012^{1}$	$0.03047^{7}$	$0.02473^{3}$	$0.02543^{4}$
	MREs	$\hat{\alpha }$	$0.25726^{3}$	$0.25514^{2}$	$0.27507^{6}$	$0.21064^{1}$	$0.27396^{5}$	$0.27666^{7}$	$0.26006^{4}$
		$\hat{\lambda }$	$0.03293^{6}$	$0.02922^{1}$	$0.03027^{4}$	$0.03036^{5}$	$0.03313^{7}$	$0.03016^{3}$	$0.02945^{2}$
		$\hat{\beta }$	$0.28995^{6}$	$0.27023^{2}$	$0.28583^{5}$	$0.23264^{1}$	$0.30681^{7}$	$0.28016^{4}$	$0.27968^{3}$
	∑Ranks		$46^{5.5}$	$15^{1}$	$46^{5.5}$	$20^{2}$	$56^{7}$	$40^{4}$	$29^{3}$
350	BIAS	$\hat{\alpha }$	$0.34345^{2}$	$0.37295^{4}$	$0.38954^{5}$	$0.24304^{1}$	$0.38986^{6}$	$0.39083^{7}$	$0.35706^{3}$
		$\hat{\lambda }$	$0.0178^{4}$	$0.01609^{1}$	$0.01784^{5}$	$0.01619^{2}$	$0.01904^{7}$	$0.01684^{3}$	$0.01839^{6}$
		$\hat{\beta }$	$0.11937^{2}$	$0.12745^{5}$	$0.14139^{6}$	$0.08654^{1}$	$0.145^{7}$	$0.12594^{3}$	$0.1262^{4}$
	MSEs	$\hat{\alpha }$	$0.14698^{2}$	$0.16367^{4}$	$0.1762^{6}$	$0.11523^{1}$	$0.17584^{5}$	$0.17771^{7}$	$0.15531^{3}$
		$\hat{\lambda }$	$0.00051^{4}$	$0.00045^{2}$	$0.00056^{5}$	$0.00043^{1}$	$0.00063^{7}$	$0.00047^{3}$	$0.00058^{6}$
		$\hat{\beta }$	$0.01941^{2}$	$0.02084^{4}$	$0.02649^{6}$	$0.01371^{1}$	$0.02779^{7}$	$0.0201^{3}$	$0.02198^{5}$
	MREs	$\hat{\alpha }$	$0.22897^{2}$	$0.24864^{4}$	$0.25969^{5}$	$0.16203^{1}$	$0.2599^{6}$	$0.26055^{7}$	$0.23804^{3}$
		$\hat{\lambda }$	$0.02374^{4}$	$0.02146^{1}$	$0.02378^{5}$	$0.02158^{2}$	$0.02539^{7}$	$0.02245^{3}$	$0.02452^{6}$
		$\hat{\beta }$	$0.23873^{2}$	$0.25491^{5}$	$0.28277^{6}$	$0.17308^{1}$	$0.29^{7}$	$0.25188^{3}$	$0.25241^{4}$
	∑Ranks		$24^{2}$	$30^{3}$	$49^{6}$	$11^{1}$	$59^{7}$	$39^{4}$	$40^{5}$

**Table 4:** Simulation results for the PMKE distribution with α=0.5, λ=1.5, and β=1.5.

*n*	Est.	Est. Par.	MLEs	ADEs	CVMEs	MPSs	LSEs	PCEs	WLSEs
20	BIAS	$\hat{\alpha }$	$0.14974^{3}$	$0.17277^{6}$	$0.17838^{7}$	$0.14762^{2}$	$0.1572^{4}$	$0.14438^{1}$	$0.16238^{5}$
		$\hat{\lambda }$	$0.3205^{7}$	$0.28181^{1}$	$0.29513^{4}$	$0.30312^{5}$	$0.29169^{3}$	$0.30945^{6}$	$0.28909^{2}$
		$\hat{\beta }$	$0.39947^{3}$	$0.4004^{4}$	$0.39723^{2}$	$0.38656^{1}$	$0.40046^{5}$	$0.41067^{7}$	$0.40114^{6}$
	MSEs	$\hat{\alpha }$	$0.03734^{3}$	$0.04755^{6}$	$0.05368^{7}$	$0.03178^{1}$	$0.03801^{4}$	$0.03377^{2}$	$0.04114^{5}$
		$\hat{\lambda }$	$0.12959^{7}$	$0.10533^{1}$	$0.11493^{4}$	$0.12076^{5}$	$0.11337^{3}$	$0.12386^{6}$	$0.1113^{2}$
		$\hat{\beta }$	$0.1831^{4}$	$0.18238^{3}$	$0.18136^{2}$	$0.1756^{1}$	$0.18355^{5}$	$0.18932^{7}$	$0.18371^{6}$
	MREs	$\hat{\alpha }$	$0.29948^{3}$	$0.34554^{6}$	$0.35675^{7}$	$0.29524^{2}$	$0.31439^{4}$	$0.28875^{1}$	$0.32475^{5}$
		$\hat{\lambda }$	$0.21367^{7}$	$0.18788^{1}$	$0.19675^{4}$	$0.20208^{5}$	$0.19446^{3}$	$0.2063^{6}$	$0.19272^{2}$
		$\hat{\beta }$	$0.26631^{3}$	$0.26693^{4}$	$0.26482^{2}$	$0.2577^{1}$	$0.26697^{5}$	$0.27378^{7}$	$0.26742^{6}$
	∑Ranks		$40^{6}$	$32^{2}$	$39^{4.5}$	$23^{1}$	$36^{3}$	$43^{7}$	$39^{4.5}$
50	BIAS	$\hat{\alpha }$	$0.12947^{1}$	$0.1473^{4}$	$0.16726^{7}$	$0.14121^{2}$	$0.15413^{6}$	$0.14132^{3}$	$0.15297^{5}$
		$\hat{\lambda }$	$0.24832^{4}$	$0.24026^{3}$	$0.24909^{5}$	$0.25179^{6}$	$0.25765^{7}$	$0.23569^{2}$	$0.23425^{1}$
		$\hat{\beta }$	$0.36268^{2}$	$0.36446^{3}$	$0.38594^{6}$	$0.35701^{1}$	$0.38617^{7}$	$0.36965^{4}$	$0.37672^{5}$
	MSEs	$\hat{\alpha }$	$0.02581^{1}$	$0.03348^{4}$	$0.04248^{7}$	$0.02857^{2}$	$0.03441^{5}$	$0.02966^{3}$	$0.03588^{6}$
		$\hat{\lambda }$	$0.0888^{6}$	$0.08048^{3}$	$0.08408^{4}$	$0.08668^{5}$	$0.09225^{7}$	$0.07847^{1}$	$0.07883^{2}$
		$\hat{\beta }$	$0.15856^{2}$	$0.16081^{3}$	$0.17706^{7}$	$0.15663^{1}$	$0.17428^{6}$	$0.16342^{4}$	$0.16961^{5}$
	MREs	$\hat{\alpha }$	$0.25894^{1}$	$0.29461^{4}$	$0.33451^{7}$	$0.28243^{2}$	$0.30826^{6}$	$0.28265^{3}$	$0.30594^{5}$
		$\hat{\lambda }$	$0.16555^{4}$	$0.16017^{3}$	$0.16606^{5}$	$0.16786^{6}$	$0.17177^{7}$	$0.15712^{2}$	$0.15617^{1}$
		$\hat{\beta }$	$0.24178^{2}$	$0.24297^{3}$	$0.26062^{7}$	$0.23801^{1}$	$0.25745^{6}$	$0.24643^{4}$	$0.25115^{5}$
	∑Ranks		$23^{1}$	$30^{4}$	$55^{6}$	$26^{2.5}$	$57^{7}$	$26^{2.5}$	$35^{5}$
100	BIAS	$\hat{\alpha }$	$0.11444^{1}$	$0.1327^{4}$	$0.14602^{7}$	$0.12568^{2}$	$0.13867^{6}$	$0.12572^{3}$	$0.13836^{5}$
		$\hat{\lambda }$	$0.19648^{3}$	$0.1892^{1}$	$0.22367^{7}$	$0.19202^{2}$	$0.21737^{6}$	$0.19741^{4}$	$0.20651^{5}$
		$\hat{\beta }$	$0.30725^{2}$	$0.33206^{4}$	$0.3784^{7}$	$0.30983^{3}$	$0.36984^{6}$	$0.30638^{1}$	$0.35393^{5}$
	MSEs	$\hat{\alpha }$	$0.0199^{1}$	$0.02695^{4}$	$0.03182^{7}$	$0.02371^{3}$	$0.02773^{5}$	$0.023^{2}$	$0.028^{6}$
		$\hat{\lambda }$	$0.05897^{4}$	$0.05214^{1}$	$0.07155^{7}$	$0.0527^{2}$	$0.06621^{6}$	$0.0548^{3}$	$0.06022^{5}$
		$\hat{\beta }$	$0.1225^{2}$	$0.13985^{4}$	$0.16835^{7}$	$0.12506^{3}$	$0.16317^{6}$	$0.12206^{1}$	$0.1524^{5}$
	MREs	$\hat{\alpha }$	$0.22889^{1}$	$0.26539^{4}$	$0.29204^{7}$	$0.25136^{2}$	$0.27733^{6}$	$0.25143^{3}$	$0.27672^{5}$
		$\hat{\lambda }$	$0.13099^{3}$	$0.12614^{1}$	$0.14912^{7}$	$0.12801^{2}$	$0.14491^{6}$	$0.13161^{4}$	$0.13767^{5}$
		$\hat{\beta }$	$0.20483^{2}$	$0.22137^{4}$	$0.25227^{7}$	$0.20656^{3}$	$0.24656^{6}$	$0.20425^{1}$	$0.23595^{5}$
	∑Ranks		$19^{1}$	$27^{4}$	$63^{7}$	$22^{2.5}$	$53^{6}$	$22^{2.5}$	$46^{5}$
200	BIAS	$\hat{\alpha }$	$0.08444^{1}$	$0.10461^{3}$	$0.11825^{6}$	$0.09634^{2}$	$0.131^{7}$	$0.10629^{4}$	$0.11375^{5}$
		$\hat{\lambda }$	$0.15163^{2}$	$0.15645^{3}$	$0.18805^{6}$	$0.1498^{1}$	$0.20784^{7}$	$0.15863^{4}$	$0.17325^{5}$
		$\hat{\beta }$	$0.2276^{1}$	$0.26839^{4}$	$0.32534^{6}$	$0.24005^{2}$	$0.33897^{7}$	$0.2612^{3}$	$0.29096^{5}$
	MSEs	$\hat{\alpha }$	$0.0119^{1}$	$0.01785^{3}$	$0.02045^{6}$	$0.0152^{2}$	$0.02539^{7}$	$0.01806^{4}$	$0.02003^{5}$
		$\hat{\lambda }$	$0.03582^{2}$	$0.03738^{3}$	$0.05181^{6}$	$0.03402^{1}$	$0.05867^{7}$	$0.03739^{4}$	$0.04404^{5}$
		$\hat{\beta }$	$0.07697^{1}$	$0.10098^{4}$	$0.13511^{6}$	$0.08518^{2}$	$0.14296^{7}$	$0.09361^{3}$	$0.11403^{5}$
	MREs	$\hat{\alpha }$	$0.16888^{1}$	$0.20921^{3}$	$0.2365^{6}$	$0.19268^{2}$	$0.262^{7}$	$0.21259^{4}$	$0.22749^{5}$
		$\hat{\lambda }$	$0.10108^{2}$	$0.1043^{3}$	$0.12537^{6}$	$0.09986^{1}$	$0.13856^{7}$	$0.10576^{4}$	$0.1155^{5}$
		$\hat{\beta }$	$0.15173^{1}$	$0.17893^{4}$	$0.2169^{6}$	$0.16003^{2}$	$0.22598^{7}$	$0.17413^{3}$	$0.19397^{5}$
	∑Ranks		$12^{1}$	$30^{3}$	$54^{6}$	$15^{2}$	$63^{7}$	$33^{4}$	$45^{5}$
350	BIAS	$\hat{\alpha }$	$0.06941^{1}$	$0.08888^{5}$	$0.10921^{7}$	$0.07805^{2}$	$0.10674^{6}$	$0.07832^{3}$	$0.08647^{4}$
		$\hat{\lambda }$	$0.11555^{2}$	$0.13707^{5}$	$0.17484^{7}$	$0.11374^{1}$	$0.17422^{6}$	$0.12827^{3}$	$0.13702^{4}$
		$\hat{\beta }$	$0.19214^{2}$	$0.23988^{5}$	$0.29714^{7}$	$0.18466^{1}$	$0.2886^{6}$	$0.19349^{3}$	$0.23249^{4}$
	MSEs	$\hat{\alpha }$	$0.00753^{1}$	$0.01261^{5}$	$0.01804^{7}$	$0.01093^{3}$	$0.01667^{6}$	$0.01026^{2}$	$0.01246^{4}$
		$\hat{\lambda }$	$0.02074^{2}$	$0.028^{4}$	$0.04453^{7}$	$0.01976^{1}$	$0.04313^{6}$	$0.02529^{3}$	$0.02867^{5}$
		$\hat{\beta }$	$0.05559^{2}$	$0.082^{5}$	$0.11691^{7}$	$0.05474^{1}$	$0.11133^{6}$	$0.05731^{3}$	$0.0783^{4}$
	MREs	$\hat{\alpha }$	$0.13881^{1}$	$0.17776^{5}$	$0.21841^{7}$	$0.1561^{2}$	$0.21348^{6}$	$0.15663^{3}$	$0.17295^{4}$
		$\hat{\lambda }$	$0.07704^{2}$	$0.09138^{5}$	$0.11656^{7}$	$0.07583^{1}$	$0.11615^{6}$	$0.08552^{3}$	$0.09135^{4}$
		$\hat{\beta }$	$0.12809^{2}$	$0.15992^{5}$	$0.1981^{7}$	$0.12311^{1}$	$0.1924^{6}$	$0.129^{3}$	$0.15499^{4}$
	∑Ranks		$15^{2}$	$44^{5}$	$63^{7}$	$13^{1}$	$54^{6}$	$26^{3}$	$37^{4}$

**Table 5:** Simulation results for the PMKE distribution with α=0.75, λ=1.5, and β=0.25.

*n*	Est.	Est. Par.	MLEs	ADEs	CVMEs	MPSs	LSEs	PCEs	WLSEs
20	BIAS	$\hat{\alpha }$	$0.20773^{1}$	$0.22369^{3}$	$0.22128^{2}$	$0.22869^{5}$	$0.24303^{6}$	$0.25651^{7}$	$0.2266^{4}$
		$\hat{\lambda }$	$0.34922^{6}$	$0.30255^{1}$	$0.33439^{4}$	$0.33596^{5}$	$0.32503^{3}$	$0.35047^{7}$	$0.31713^{2}$
		$\hat{\beta }$	$0.09238^{4}$	$0.0795^{1}$	$0.09025^{3}$	$0.09555^{5}$	$0.09746^{6}$	$0.12761^{7}$	$0.08111^{2}$
	MSEs	$\hat{\alpha }$	$0.05242^{1}$	$0.05785^{3}$	$0.05756^{2}$	$0.06355^{5}$	$0.06916^{6}$	$0.07602^{7}$	$0.05886^{4}$
		$\hat{\lambda }$	$0.1526^{7}$	$0.12083^{1}$	$0.14254^{5}$	$0.13906^{4}$	$0.13558^{3}$	$0.14711^{6}$	$0.12841^{2}$
		$\hat{\beta }$	$0.0144^{5}$	$0.01056^{1}$	$0.013^{3}$	$0.01423^{4}$	$0.01466^{6}$	$0.02212^{7}$	$0.01102^{2}$
	MREs	$\hat{\alpha }$	$0.27697^{1}$	$0.29825^{3}$	$0.29504^{2}$	$0.30493^{5}$	$0.32404^{6}$	$0.35535^{7}$	$0.30213^{4}$
		$\hat{\lambda }$	$0.23282^{6}$	$0.2017^{1}$	$0.22293^{4}$	$0.22397^{5}$	$0.21668^{3}$	$0.23365^{7}$	$0.21142^{2}$
		$\hat{\beta }$	$0.36953^{4}$	$0.31802^{1}$	$0.36098^{3}$	$0.38221^{5}$	$0.38985^{6}$	$0.51044^{7}$	$0.32442^{2}$
	∑Ranks		$35^{4}$	$15^{1}$	$28^{3}$	$43^{5}$	$45^{6}$	$62^{7}$	$24^{2}$
50	BIAS	$\hat{\alpha }$	$0.19535^{1}$	$0.21235^{4}$	$0.21614^{5}$	$0.20562^{2}$	$0.22003^{6}$	$0.24855^{7}$	$0.20956^{3}$
		$\hat{\lambda }$	$0.29561^{4}$	$0.26051^{1}$	$0.31241^{6}$	$0.28479^{2}$	$0.3087^{5}$	$0.34083^{7}$	$0.29357^{3}$
		$\hat{\beta }$	$0.08022^{5}$	$0.07184^{3}$	$0.07913^{4}$	$0.0712^{1}$	$0.08145^{6}$	$0.10378^{7}$	$0.07175^{2}$
	MSEs	$\hat{\alpha }$	$0.0463^{1}$	$0.05187^{4}$	$0.05327^{5}$	$0.04948^{2}$	$0.0556^{6}$	$0.06784^{7}$	$0.05105^{3}$
		$\hat{\lambda }$	$0.11949^{4}$	$0.09376^{1}$	$0.1264^{6}$	$0.1053^{2}$	$0.12321^{5}$	$0.14317^{7}$	$0.11306^{3}$
		$\hat{\beta }$	$0.01036^{6}$	$0.00786^{3}$	$0.00955^{4}$	$0.00713^{1}$	$0.00985^{5}$	$0.01533^{7}$	$0.0078^{2}$
	MREs	$\hat{\alpha }$	$0.26047^{1}$	$0.28314^{4}$	$0.28819^{5}$	$0.27416^{2}$	$0.29338^{6}$	$0.3314^{7}$	$0.27942^{3}$
		$\hat{\lambda }$	$0.19708^{4}$	$0.17367^{1}$	$0.20827^{6}$	$0.18986^{2}$	$0.2058^{5}$	$0.22722^{7}$	$0.19572^{3}$
		$\hat{\beta }$	$0.32089^{5}$	$0.28738^{3}$	$0.31653^{4}$	$0.28479^{1}$	$0.32579^{6}$	$0.41513^{7}$	$0.28701^{2}$
	∑Ranks		$31^{4}$	$24^{2.5}$	$45^{5}$	$15^{1}$	$50^{6}$	$63^{7}$	$24^{2.5}$
100	BIAS	$\hat{\alpha }$	$0.17146^{1}$	$0.18852^{3}$	$0.19665^{5}$	$0.18471^{2}$	$0.20589^{6}$	$0.24556^{7}$	$0.19053^{4}$
		$\hat{\lambda }$	$0.26301^{3}$	$0.24329^{2}$	$0.29034^{6}$	$0.23282^{1}$	$0.27692^{5}$	$0.32626^{7}$	$0.26575^{4}$
		$\hat{\beta }$	$0.0627^{3}$	$0.06024^{1}$	$0.07468^{6}$	$0.06058^{2}$	$0.07285^{5}$	$0.08418^{7}$	$0.06609^{4}$
	MSEs	$\hat{\alpha }$	$0.03716^{1}$	$0.04253^{3}$	$0.04528^{5}$	$0.04197^{2}$	$0.049^{6}$	$0.065^{7}$	$0.04358^{4}$
		$\hat{\lambda }$	$0.09625^{3.5}$	$0.08068^{2}$	$0.11197^{6}$	$0.07366^{1}$	$0.10194^{5}$	$0.13469^{7}$	$0.09625^{3.5}$
		$\hat{\beta }$	$0.00601^{3}$	$0.00511^{1}$	$0.00783^{6}$	$0.00518^{2}$	$0.00749^{5}$	$0.00994^{7}$	$0.00639^{4}$
	MREs	$\hat{\alpha }$	$0.22861^{1}$	$0.25136^{3}$	$0.26221^{5}$	$0.24628^{2}$	$0.27452^{6}$	$0.32741^{7}$	$0.25404^{4}$
		$\hat{\lambda }$	$0.17534^{3}$	$0.16219^{2}$	$0.19356^{6}$	$0.15521^{1}$	$0.18461^{5}$	$0.21751^{7}$	$0.17716^{4}$
		$\hat{\beta }$	$0.2508^{3}$	$0.24096^{1}$	$0.29872^{6}$	$0.24231^{2}$	$0.29142^{5}$	$0.3367^{7}$	$0.26437^{4}$
	∑Ranks		$21.5^{3}$	$18^{2}$	$51^{6}$	$15^{1}$	$48^{5}$	$63^{7}$	$35.5^{4}$
200	BIAS	$\hat{\alpha }$	$0.14956^{1}$	$0.16078^{3}$	$0.1828^{6}$	$0.15529^{2}$	$0.17908^{5}$	$0.22997^{7}$	$0.16405^{4}$
		$\hat{\lambda }$	$0.20387^{2}$	$0.20984^{3}$	$0.25381^{6}$	$0.19687^{1}$	$0.24438^{5}$	$0.30728^{7}$	$0.22585^{4}$
		$\hat{\beta }$	$0.05144^{2}$	$0.05281^{3}$	$0.06348^{6}$	$0.04901^{1}$	$0.06305^{5}$	$0.07363^{7}$	$0.05517^{4}$
	MSEs	$\hat{\alpha }$	$0.02958^{1}$	$0.03329^{3}$	$0.04027^{6}$	$0.03177^{2}$	$0.03953^{5}$	$0.05772^{7}$	$0.03398^{4}$
		$\hat{\lambda }$	$0.06103^{2}$	$0.0625^{3}$	$0.08901^{6}$	$0.05646^{1}$	$0.08353^{5}$	$0.12265^{7}$	$0.07255^{4}$
		$\hat{\beta }$	$0.00398^{2.5}$	$0.00398^{2.5}$	$0.00557^{5}$	$0.00352^{1}$	$0.0056^{6}$	$0.00769^{7}$	$0.00429^{4}$
	MREs	$\hat{\alpha }$	$0.19942^{1}$	$0.21438^{3}$	$0.24374^{6}$	$0.20705^{2}$	$0.23877^{5}$	$0.30662^{7}$	$0.21873^{4}$
		$\hat{\lambda }$	$0.13592^{2}$	$0.13989^{3}$	$0.16921^{6}$	$0.13124^{1}$	$0.16292^{5}$	$0.20485^{7}$	$0.15057^{4}$
		$\hat{\beta }$	$0.20575^{2}$	$0.21123^{3}$	$0.25391^{6}$	$0.19606^{1}$	$0.25221^{5}$	$0.29452^{7}$	$0.22066^{4}$
	∑Ranks		$15.5^{2}$	$26.5^{3}$	$53^{6}$	$12^{1}$	$46^{5}$	$63^{7}$	$36^{4}$
350	BIAS	$\hat{\alpha }$	$0.12478^{1}$	$0.14793^{3}$	$0.15803^{5}$	$0.12955^{2}$	$0.16534^{6}$	$0.22432^{7}$	$0.15062^{4}$
		$\hat{\lambda }$	$0.16581^{2}$	$0.19051^{3}$	$0.22704^{5}$	$0.14927^{1}$	$0.23644^{6}$	$0.29277^{7}$	$0.19926^{4}$
		$\hat{\beta }$	$0.04055^{2}$	$0.04831^{3}$	$0.05566^{5}$	$0.03872^{1}$	$0.05647^{6}$	$0.06422^{7}$	$0.04911^{4}$
	MSEs	$\hat{\alpha }$	$0.02135^{1}$	$0.02916^{3}$	$0.03265^{5}$	$0.02425^{2}$	$0.03442^{6}$	$0.05524^{7}$	$0.02995^{4}$
		$\hat{\lambda }$	$0.04211^{2}$	$0.05268^{3}$	$0.07368^{5}$	$0.033^{1}$	$0.07872^{6}$	$0.11143^{7}$	$0.05687^{4}$
		$\hat{\beta }$	$0.00249^{2}$	$0.00333^{3}$	$0.00435^{5}$	$0.00213^{1}$	$0.00447^{6}$	$0.0055^{7}$	$0.00342^{4}$
	MREs	$\hat{\alpha }$	$0.16637^{1}$	$0.19725^{3}$	$0.21071^{5}$	$0.17274^{2}$	$0.22045^{6}$	$0.29909^{7}$	$0.20083^{4}$
		$\hat{\lambda }$	$0.11054^{2}$	$0.12701^{3}$	$0.15136^{5}$	$0.09951^{1}$	$0.15763^{6}$	$0.19518^{7}$	$0.13284^{4}$
		$\hat{\beta }$	$0.16221^{2}$	$0.19324^{3}$	$0.22264^{5}$	$0.15488^{1}$	$0.22588^{6}$	$0.25689^{7}$	$0.19645^{4}$
	∑Ranks		$15^{2}$	$27^{3}$	$45^{5}$	$12^{1}$	$54^{6}$	$63^{7}$	$36^{4}$

**Table 6:** Simulation results for the PMKE distribution with α=0.75, λ=0.5, and β=2.5.

*n*	Est.	Est. Par.	MLEs	ADEs	CVMEs	MPSs	LSEs	PCEs	WLSEs
20	BIAS	$\hat{\alpha }$	$0.12497^{2}$	$0.12865^{5}$	$0.13515^{7}$	$0.12258^{1}$	$0.13143^{6}$	$0.12533^{3}$	$0.12861^{4}$
		$\hat{\lambda }$	$0.08677^{2}$	$0.08903^{5}$	$0.097^{7}$	$0.08305^{1}$	$0.09674^{6}$	$0.08721^{3}$	$0.08767^{4}$
		$\hat{\beta }$	$0.28943^{7}$	$0.19862^{3}$	$0.20827^{4}$	$0.17409^{1}$	$0.22532^{5}$	$0.2395^{6}$	$0.18915^{2}$
	MSEs	$\hat{\alpha }$	$0.02213^{2.5}$	$0.02304^{4}$	$0.02521^{7}$	$0.02116^{1}$	$0.02401^{6}$	$0.02213^{2.5}$	$0.02323^{5}$
		$\hat{\lambda }$	$0.01189^{3}$	$0.01221^{5}$	$0.01415^{7}$	$0.01065^{1}$	$0.01407^{6}$	$0.0117^{2}$	$0.01212^{4}$
		$\hat{\beta }$	$0.0392^{6}$	$0.03834^{4}$	$0.03896^{5}$	$0.03381^{1}$	$0.03929^{7}$	$0.03824^{3}$	$0.03707^{2}$
	MREs	$\hat{\alpha }$	$0.16663^{2}$	$0.17153^{5}$	$0.1802^{7}$	$0.16344^{1}$	$0.17524^{6}$	$0.1671^{3}$	$0.17148^{4}$
		$\hat{\lambda }$	$0.17353^{2}$	$0.17806^{5}$	$0.19401^{7}$	$0.1661^{1}$	$0.19348^{6}$	$0.17442^{3}$	$0.17533^{4}$
		$\hat{\beta }$	$0.09577^{7}$	$0.0785^{5}$	$0.07731^{4}$	$0.06963^{1}$	$0.07135^{2}$	$0.0794^{6}$	$0.07566^{3}$
	∑Ranks		$33.5^{4}$	$41^{5}$	$55^{7}$	$9^{1}$	$50^{6}$	$31.5^{2}$	$32^{3}$
50	BIAS	$\hat{\alpha }$	$0.09107^{3}$	$0.09413^{4}$	$0.09963^{6}$	$0.08776^{1}$	$0.10032^{7}$	$0.08983^{2}$	$0.09796^{5}$
		$\hat{\lambda }$	$0.05438^{2}$	$0.05921^{4}$	$0.06494^{7}$	$0.05306^{1}$	$0.06307^{6}$	$0.05604^{3}$	$0.06154^{5}$
		$\hat{\beta }$	$0.19039^{6}$	$0.18917^{3}$	$0.18949^{4}$	$0.1665^{1}$	$0.18996^{5}$	$0.19087^{7}$	$0.18801^{2}$
	MSEs	$\hat{\alpha }$	$0.01252^{3}$	$0.01343^{4}$	$0.01492^{7}$	$0.01145^{1}$	$0.01488^{6}$	$0.01232^{2}$	$0.01447^{5}$
		$\hat{\lambda }$	$0.0048^{2}$	$0.00565^{4}$	$0.00668^{7}$	$0.00462^{1}$	$0.00632^{6}$	$0.00506^{3}$	$0.00597^{5}$
		$\hat{\beta }$	$0.0374^{6}$	$0.03709^{3}$	$0.03726^{4}$	$0.0326^{1}$	$0.03737^{5}$	$0.03747^{7}$	$0.0368^{2}$
	MREs	$\hat{\alpha }$	$0.12142^{3}$	$0.12551^{4}$	$0.13283^{6}$	$0.11702^{1}$	$0.13375^{7}$	$0.11978^{2}$	$0.13061^{5}$
		$\hat{\lambda }$	$0.10876^{2}$	$0.11842^{4}$	$0.12988^{7}$	$0.10611^{1}$	$0.12613^{6}$	$0.11209^{3}$	$0.12308^{5}$
		$\hat{\beta }$	$0.07616^{6}$	$0.07567^{3}$	$0.0758^{4}$	$0.0666^{1}$	$0.07599^{5}$	$0.07635^{7}$	$0.0752^{2}$
	∑Ranks		$33^{2.5}$	$33^{2.5}$	$52^{6}$	$9^{1}$	$53^{7}$	$36^{4.5}$	$36^{4.5}$
100	BIAS	$\hat{\alpha }$	$0.0749^{2}$	$0.07735^{5}$	$0.08385^{7}$	$0.06886^{1}$	$0.07891^{6}$	$0.07579^{3}$	$0.07639^{4}$
		$\hat{\lambda }$	$0.03988^{2}$	$0.04416^{5}$	$0.04588^{6}$	$0.03897^{1}$	$0.04595^{7}$	$0.04122^{3}$	$0.04357^{4}$
		$\hat{\beta }$	$0.18383^{2}$	$0.18649^{4}$	$0.18863^{7}$	$0.15642^{1}$	$0.18705^{6}$	$0.18606^{3}$	$0.18699^{5}$
	MSEs	$\hat{\alpha }$	$0.0084^{2}$	$0.00899^{5}$	$0.01058^{7}$	$0.00723^{1}$	$0.00946^{6}$	$0.00875^{3}$	$0.00887^{4}$
		$\hat{\lambda }$	$0.00255^{2}$	$0.00302^{5}$	$0.00335^{7}$	$0.00249^{1}$	$0.00331^{6}$	$0.00269^{3}$	$0.00296^{4}$
		$\hat{\beta }$	$0.03572^{2}$	$0.03653^{4}$	$0.03699^{6}$	$0.03062^{1}$	$0.03702^{7}$	$0.03623^{3}$	$0.03657^{5}$
	MREs	$\hat{\alpha }$	$0.09987^{2}$	$0.10314^{5}$	$0.1118^{7}$	$0.09181^{1}$	$0.10521^{6}$	$0.10106^{3}$	$0.10186^{4}$
		$\hat{\lambda }$	$0.07975^{2}$	$0.08831^{5}$	$0.09176^{6}$	$0.07794^{1}$	$0.09191^{7}$	$0.08243^{3}$	$0.08714^{4}$
		$\hat{\beta }$	$0.07353^{2}$	$0.0746^{4}$	$0.07545^{7}$	$0.06257^{1}$	$0.07503^{6}$	$0.07443^{3}$	$0.0748^{5}$
	∑Ranks		$18^{2}$	$42^{5}$	$60^{7}$	$9^{1}$	$57^{6}$	$27^{3}$	$39^{4}$
200	BIAS	$\hat{\alpha }$	$0.06214^{2}$	$0.06538^{5}$	$0.06551^{6}$	$0.05398^{1}$	$0.06632^{7}$	$0.06473^{3}$	$0.06479^{4}$
		$\hat{\lambda }$	$0.02942^{2}$	$0.03183^{3}$	$0.03487^{7}$	$0.02814^{1}$	$0.03414^{6}$	$0.03203^{4}$	$0.03345^{5}$
		$\hat{\beta }$	$0.17817^{2}$	$0.18069^{4}$	$0.18661^{7}$	$0.13861^{1}$	$0.18611^{6}$	$0.18015^{3}$	$0.1837^{5}$
	MSEs	$\hat{\alpha }$	$0.00565^{2}$	$0.00634^{6}$	$0.00632^{5}$	$0.00451^{1}$	$0.00654^{7}$	$0.00613^{3}$	$0.00626^{4}$
		$\hat{\lambda }$	$0.00138^{2}$	$0.00158^{3}$	$0.00189^{7}$	$0.00125^{1}$	$0.00178^{6}$	$0.00161^{4}$	$0.00172^{5}$
		$\hat{\beta }$	$0.03426^{2}$	$0.03489^{4}$	$0.03644^{7}$	$0.0271^{1}$	$0.03639^{6}$	$0.03482^{3}$	$0.03564^{5}$
	MREs	$\hat{\alpha }$	$0.08285^{2}$	$0.08717^{5}$	$0.08735^{6}$	$0.07197^{1}$	$0.08842^{7}$	$0.0863^{3}$	$0.08638^{4}$
		$\hat{\lambda }$	$0.05884^{2}$	$0.06365^{3}$	$0.06973^{7}$	$0.05629^{1}$	$0.06828^{6}$	$0.06406^{4}$	$0.06689^{5}$
		$\hat{\beta }$	$0.07127^{2}$	$0.07228^{4}$	$0.07465^{7}$	$0.05544^{1}$	$0.07445^{6}$	$0.07206^{3}$	$0.07348^{5}$
	∑Ranks		$18^{2}$	$37^{4}$	$59^{7}$	$9^{1}$	$57^{6}$	$30^{3}$	$42^{5}$
350	BIAS	$\hat{\alpha }$	$0.05629^{2}$	$0.06045^{7}$	$0.05957^{3}$	$0.04776^{1}$	$0.06003^{6}$	$0.05989^{5}$	$0.05968^{4}$
		$\hat{\lambda }$	$0.02347^{2}$	$0.02658^{5}$	$0.02796^{6}$	$0.02201^{1}$	$0.02866^{7}$	$0.02574^{3}$	$0.02654^{4}$
		$\hat{\beta }$	$0.17262^{2}$	$0.17817^{5}$	$0.18349^{6}$	$0.11708^{1}$	$0.18369^{7}$	$0.17465^{3}$	$0.17787^{4}$
	MSEs	$\hat{\alpha }$	$0.00435^{2}$	$0.00492^{4}$	$0.00501^{7}$	$0.00344^{1}$	$0.00497^{6}$	$0.00494^{5}$	$0.00486^{3}$
		$\hat{\lambda }$	$0.00084^{2}$	$0.00107^{5}$	$0.00119^{6}$	$0.00075^{1}$	$0.00123^{7}$	$0.00101^{3}$	$0.00104^{4}$
		$\hat{\beta }$	$0.03267^{2}$	$0.03419^{5}$	$0.0356^{6}$	$0.02274^{1}$	$0.03562^{7}$	$0.0333^{3}$	$0.03413^{4}$
	MREs	$\hat{\alpha }$	$0.07506^{2}$	$0.0806^{7}$	$0.07943^{3}$	$0.06368^{1}$	$0.08003^{6}$	$0.07986^{5}$	$0.07957^{4}$
		$\hat{\lambda }$	$0.04693^{2}$	$0.05315^{5}$	$0.05593^{6}$	$0.04402^{1}$	$0.05731^{7}$	$0.05148^{3}$	$0.05308^{4}$
		$\hat{\beta }$	$0.06905^{2}$	$0.07127^{5}$	$0.0734^{6}$	$0.04683^{1}$	$0.07348^{7}$	$0.06986^{3}$	$0.07115^{4}$
	∑Ranks		$18^{2}$	$48^{5}$	$49^{6}$	$9^{1}$	$60^{7}$	$33^{3}$	$35^{4}$

**Table 7:** Partial and overall ranks of all estimation methods for the PMKE distribution.

Parameter	*n*	MLEs	ADEs	CVMEs	MPSEs	LSEs	PCEs	WLSEs
α=0.25,λ=0.5,β=0.75	20	1	3	7	2	5	6	4
	50	1	3	5.5	2	7	5.5	4
	100	1	3	7	2	5	6	4
	200	1	3	6	2	7	5	4
	350	2	3	5	1	7	6	4
α=1.5,λ=0.75,β=0.5	20	6.5	3	6.5	1	4.5	4.5	2
	50	6	2	5	1	7	4	3
	100	5	3	7	1	4	6	2
	200	5.5	1	5.5	2	7	4	3
	350	2	3	6	1	7	4	5
α=0.5,λ=1.5,β=1.5	20	6	2	4.5	1	3	7	4.5
	50	1	4	6	2.5	7	2.5	5
	100	1	4	7	2.5	6	2.5	5
	200	1	3	6	2	7	4	5
	350	2	5	7	1	6	3	4
α=0.75,λ=1.5,β=0.25	20	4	1	3	5	6	7	2
	50	4	2.5	5	1	6	7	2.5
	100	3	2	6	1	5	7	4
	200	2	3	6	1	5	7	4
	350	2	3	5	1	6	7	4
α=0.75,λ=0.5,β=2.5	20	4	5	7	1	6	2	3
	50	2.5	2.5	6	1	7	4.5	4.5
	100	2	5	7	1	6	3	4
	200	2	4	7	1	6	3	5
	350	2	5	6	1	7	3	4
∑ Ranks		69.5	78	149	38	149.5	120.5	95.5
Overall Rank		2	3	6	1	7	5	4

**Table 8:** Simulation results for the PMKE distribution with (α=0.5,λ=0.25,β=0.75) and (α=1.5,λ=0.75,β=0.5).

*n*	Est.	Est. Par.	BSE	BLN	BGE	BSE	BLN	BGE
			α=0.5,λ=0.25,β=0.75			α=1.5,λ=0.75,β=0.5		
20	BIAS	$\hat{\alpha }$	$0.0868^{3}$	$0.08283^{2}$	$0.07946^{1}$	$0.17885^{2}$	$0.1744^{1}$	$0.17938^{3}$
		$\hat{\lambda }$	$0.19586^{3}$	$0.16718^{2}$	$0.15811^{1}$	$0.03939^{1}$	$0.03963^{2.5}$	$0.03963^{2.5}$
		$\hat{\beta }$	$0.26146^{2}$	$0.26102^{1}$	$0.26858^{3}$	$0.04281^{3}$	$0.04015^{1}$	$0.04033^{2}$
	MSEs	$\hat{\alpha }$	$0.01081^{3}$	$0.00813^{2}$	$0.00755^{1}$	$0.09086^{3}$	$0.07436^{1}$	$0.07971^{2}$
		$\hat{\lambda }$	$0.07729^{3}$	$0.04846^{2}$	$0.04357^{1}$	$0.00185^{3}$	$0.00184^{1.5}$	$0.00184^{1.5}$
		$\hat{\beta }$	$0.08043^{3}$	$0.07067^{1}$	$0.07464^{2}$	$0.0025^{3}$	$0.00203^{1}$	$0.00205^{2}$
	MREs	$\hat{\alpha }$	$0.09109^{1}$	$0.33131^{3}$	$0.31784^{2}$	$0.03223^{1}$	$0.11626^{2}$	$0.11959^{3}$
		$\hat{\lambda }$	$0.08534^{1}$	$0.33436^{3}$	$0.31622^{2}$	$0.05182^{1}$	$0.05284^{2.5}$	$0.05284^{2.5}$
		$\hat{\beta }$	$0.05723^{1}$	$0.34802^{2}$	$0.3581^{3}$	$0.08^{1}$	$0.08029^{2}$	$0.08066^{3}$
	∑Ranks		$20^{3}$	$18^{2}$	$16^{1}$	$18^{2}$	$14.5^{1}$	$21.5^{3}$
50	BIAS	$\hat{\alpha }$	$0.06153^{3}$	$0.05668^{2}$	$0.05495^{1}$	$0.16494^{3}$	$0.16099^{1}$	$0.16335^{2}$
		$\hat{\lambda }$	$0.13328^{3}$	$0.11735^{2}$	$0.11356^{1}$	$0.0313^{1}$	$0.03149^{3}$	$0.03148^{2}$
		$\hat{\beta }$	$0.15785^{2}$	$0.15435^{1}$	$0.15831^{3}$	$0.04167^{3}$	$0.03816^{2}$	$0.038^{1}$
	MSEs	$\hat{\alpha }$	$0.00613^{3}$	$0.00467^{2}$	$0.00442^{1}$	$0.06401^{3}$	$0.05357^{1}$	$0.05558^{2}$
		$\hat{\lambda }$	$0.03111^{3}$	$0.02073^{2}$	$0.01948^{1}$	$0.00132^{2}$	$0.00132^{2}$	$0.00132^{2}$
		$\hat{\beta }$	$0.034^{3}$	$0.02787^{1}$	$0.02925^{2}$	$0.00258^{3}$	$0.002^{2}$	$0.00197^{1}$
	MREs	$\hat{\alpha }$	$0.09002^{1}$	$0.22672^{3}$	$0.21981^{2}$	$0.02861^{1}$	$0.10733^{2}$	$0.1089^{3}$
		$\hat{\lambda }$	$0.08316^{1}$	$0.2347^{3}$	$0.22713^{2}$	$0.04079^{1}$	$0.04198^{2.5}$	$0.04198^{2.5}$
		$\hat{\beta }$	$0.05429^{1}$	$0.2058^{2}$	$0.21108^{3}$	$0.07513^{1}$	$0.07632^{3}$	$0.07601^{2}$
	∑Ranks		$20^{3}$	$18^{2}$	$16^{1}$	$18^{2}$	$18.5^{3}$	$17.5^{1}$
100	BIAS	$\hat{\alpha }$	$0.04085^{3}$	$0.03729^{2}$	$0.03653^{1}$	$0.12322^{3}$	$0.11728^{1}$	$0.11829^{2}$
		$\hat{\lambda }$	$0.08381^{3}$	$0.07405^{2}$	$0.07252^{1}$	$0.02522^{1}$	$0.02546^{2.5}$	$0.02546^{2.5}$
		$\hat{\beta }$	$0.09564^{3}$	$0.09046^{1}$	$0.09208^{2}$	$0.03416^{3}$	$0.03141^{2}$	$0.03127^{1}$
	MSEs	$\hat{\alpha }$	$0.00272^{3}$	$0.00207^{2}$	$0.00198^{1}$	$0.0328^{3}$	$0.02692^{1}$	$0.02757^{2}$
		$\hat{\lambda }$	$0.01268^{3}$	$0.00874^{2}$	$0.00834^{1}$	$0.00092^{1}$	$0.00093^{2.5}$	$0.00093^{2.5}$
		$\hat{\beta }$	$0.01381^{3}$	$0.01086^{1}$	$0.01124^{2}$	$0.0019^{3}$	$0.0015^{2}$	$0.00147^{1}$
	MREs	$\hat{\alpha }$	$0.07858^{1}$	$0.14915^{3}$	$0.14612^{2}$	$0.02918^{1}$	$0.07818^{2}$	$0.07886^{3}$
		$\hat{\lambda }$	$0.07506^{1}$	$0.1481^{3}$	$0.14504^{2}$	$0.03385^{1}$	$0.03394^{2.5}$	$0.03394^{2.5}$
		$\hat{\beta }$	$0.05148^{1}$	$0.12061^{2}$	$0.12278^{3}$	$0.06197^{1}$	$0.06283^{3}$	$0.06254^{2}$
	∑Ranks		$21^{3}$	$18^{2}$	$15^{1}$	$17^{1}$	$18.5^{2.5}$	$18.5^{2.5}$
200	BIAS	$\hat{\alpha }$	$0.0263^{3}$	$0.02379^{2}$	$0.02357^{1}$	$0.08503^{3}$	$0.07831^{1}$	$0.07864^{2}$
		$\hat{\lambda }$	$0.05495^{3}$	$0.04845^{2}$	$0.04819^{1}$	$0.01865^{1}$	$0.01879^{2.5}$	$0.01879^{2.5}$
		$\hat{\beta }$	$0.05416^{3}$	$0.04784^{1}$	$0.04826^{2}$	$0.02693^{3}$	$0.02625^{2}$	$0.0262^{1}$
	MSEs	$\hat{\alpha }$	$0.00115^{3}$	$0.0009^{2}$	$0.00088^{1}$	$0.01516^{3}$	$0.01255^{1}$	$0.0127^{2}$
		$\hat{\lambda }$	$0.00479^{3}$	$0.00357^{2}$	$0.00352^{1}$	$0.00057^{2}$	$0.00057^{2}$	$0.00057^{2}$
		$\hat{\beta }$	$0.00468^{3}$	$0.00359^{1}$	$0.00365^{2}$	$0.00125^{3}$	$0.0011^{2}$	$0.00109^{1}$
	MREs	$\hat{\alpha }$	$0.07635^{1}$	$0.09517^{3}$	$0.09428^{2}$	$0.03063^{1}$	$0.0522^{2}$	$0.05243^{3}$
		$\hat{\lambda }$	$0.07401^{1}$	$0.09689^{3}$	$0.09639^{2}$	$0.02443^{1}$	$0.02505^{2.5}$	$0.02505^{2.5}$
		$\hat{\beta }$	$0.05199^{1}$	$0.06378^{2}$	$0.06435^{3}$	$0.04583^{1}$	$0.05251^{3}$	$0.05241^{2}$
	∑Ranks		$21^{3}$	$18^{2}$	$15^{1}$	$18^{2}$	$18^{2}$	$18^{2}$
350	BIAS	$\hat{\alpha }$	$0.01921^{3}$	$0.01808^{2}$	$0.01807^{1}$	$0.08388^{3}$	$0.08073^{1}$	$0.08102^{2}$
		$\hat{\lambda }$	$0.04376^{3}$	$0.04079^{2}$	$0.04069^{1}$	$0.01651^{1}$	$0.01657^{2.5}$	$0.01657^{2.5}$
		$\hat{\beta }$	$0.04661^{3}$	$0.04486^{1}$	$0.04492^{2}$	$0.02716^{3}$	$0.02658^{2}$	$0.02645^{1}$
	MSEs	$\hat{\alpha }$	$0.00058^{3}$	$0.0005^{1.5}$	$0.0005^{1.5}$	$0.01486^{3}$	$0.01288^{1}$	$0.01303^{2}$
		$\hat{\lambda }$	$0.00299^{3}$	$0.00235^{2}$	$0.00233^{1}$	$0.0004^{2}$	$0.0004^{2}$	$0.0004^{2}$
		$\hat{\beta }$	$0.00344^{3}$	$0.00293^{1.5}$	$0.00293^{1.5}$	$0.00154^{3}$	$0.00127^{2}$	$0.00124^{1}$
	MREs	$\hat{\alpha }$	$0.07056^{1}$	$0.0723^{3}$	$0.07226^{2}$	$0.02957^{1}$	$0.05382^{2}$	$0.05401^{3}$
		$\hat{\lambda }$	$0.06574^{1}$	$0.08158^{3}$	$0.08139^{2}$	$0.0218^{1}$	$0.0221^{2.5}$	$0.0221^{2.5}$
		$\hat{\beta }$	$0.04356^{1}$	$0.05982^{2}$	$0.0599^{3}$	$0.04325^{1}$	$0.05316^{3}$	$0.0529^{2}$
	∑Ranks		$21^{3}$	$18^{2}$	$15^{1}$	$18^{2}$	$18^{2}$	$18^{2}$

**Table 9:** Simulation results for the PMKE distribution with (α=0.5,λ=1.5,β=1.5) and (α=0.75,λ=1.5,β=0.25).

*n*	Est.	Est. Par.	BSE	BLN	BGE	BSE	BLN	BGE
			α=0.5,λ=1.5,β=1.5			α=0.75,λ=1.5,β=0.25		
20	BIAS	$\hat{\alpha }$	$0.04431^{3}$	$0.04253^{1}$	$0.04267^{2}$	$0.06041^{3}$	$0.05806^{1}$	$0.05882^{2}$
		$\hat{\lambda }$	$0.06081^{3}$	$0.0582^{1}$	$0.05825^{2}$	$0.06484^{3}$	$0.06182^{1}$	$0.0619^{2}$
		$\hat{\beta }$	$0.14282^{3}$	$0.13908^{1}$	$0.14166^{2}$	$0.02584^{3}$	$0.02558^{2}$	$0.0255^{1}$
	MSEs	$\hat{\alpha }$	$0.0028^{3}$	$0.00247^{1}$	$0.00249^{2}$	$0.00685^{3}$	$0.0055^{1}$	$0.00577^{2}$
		$\hat{\lambda }$	$0.00625^{3}$	$0.0052^{1}$	$0.00522^{2}$	$0.0081^{3}$	$0.00677^{1}$	$0.00683^{2}$
		$\hat{\beta }$	$0.04973^{3}$	$0.03877^{1}$	$0.04098^{2}$	$0.00102^{3}$	$0.00094^{2}$	$0.00093^{1}$
	MREs	$\hat{\alpha }$	$0.07672^{1}$	$0.08506^{2}$	$0.08533^{3}$	$0.05682^{1}$	$0.07742^{2}$	$0.07843^{3}$
		$\hat{\lambda }$	$0.03137^{1}$	$0.0388^{2}$	$0.03883^{3}$	$0.03255^{1}$	$0.04121^{2}$	$0.04127^{3}$
		$\hat{\beta }$	$0.03024^{1}$	$0.09272^{2}$	$0.09444^{3}$	$0.08948^{1}$	$0.10231^{3}$	$0.102^{2}$
	∑Ranks		$21^{2.5}$	$12^{1}$	$21^{2.5}$	$21^{3}$	$15^{1}$	$18^{2}$
50	BIAS	$\hat{\alpha }$	$0.03437^{3}$	$0.03281^{1}$	$0.03283^{2}$	$0.04877^{3}$	$0.04694^{1}$	$0.04713^{2}$
		$\hat{\lambda }$	$0.0516^{3}$	$0.05087^{1.5}$	$0.05087^{1.5}$	$0.05331^{3}$	$0.05163^{1.5}$	$0.05163^{1.5}$
		$\hat{\beta }$	$0.0836^{3}$	$0.07722^{1}$	$0.07783^{2}$	$0.02281^{3}$	$0.02266^{2}$	$0.02263^{1}$
	MSEs	$\hat{\alpha }$	$0.00176^{3}$	$0.00154^{1.5}$	$0.00154^{1.5}$	$0.00387^{3}$	$0.00332^{1}$	$0.00337^{2}$
		$\hat{\lambda }$	$0.00383^{3}$	$0.00339^{1.5}$	$0.00339^{1.5}$	$0.00394^{3}$	$0.00337^{1.5}$	$0.00337^{1.5}$
		$\hat{\beta }$	$0.02047^{3}$	$0.01759^{1}$	$0.01824^{2}$	$0.00082^{3}$	$0.00078^{1.5}$	$0.00078^{1.5}$
	MREs	$\hat{\alpha }$	$0.06135^{1}$	$0.06563^{2}$	$0.06566^{3}$	$0.05437^{1}$	$0.06259^{2}$	$0.06284^{3}$
		$\hat{\lambda }$	$0.02958^{1}$	$0.03391^{2.5}$	$0.03391^{2.5}$	$0.029^{1}$	$0.03442^{2.5}$	$0.03442^{2.5}$
		$\hat{\beta }$	$0.03096^{1}$	$0.05148^{2}$	$0.05189^{3}$	$0.08374^{1}$	$0.09062^{3}$	$0.09051^{2}$
	∑Ranks		$21^{3}$	$14^{1}$	$19^{2}$	$21^{3}$	$16^{1}$	$17^{2}$
100	BIAS	$\hat{\alpha }$	$0.02929^{3}$	$0.02892^{1.5}$	$0.02892^{1.5}$	$0.04333^{3}$	$0.0425^{1}$	$0.04254^{2}$
		$\hat{\lambda }$	$0.048^{1}$	$0.0481^{2.5}$	$0.0481^{2.5}$	$0.04399^{3}$	$0.04308^{1.5}$	$0.04308^{1.5}$
		$\hat{\beta }$	$0.05484^{3}$	$0.05173^{1}$	$0.05178^{2}$	$0.01975^{3}$	$0.01954^{2}$	$0.01953^{1}$
	MSEs	$\hat{\alpha }$	$0.0012^{3}$	$0.00115^{1.5}$	$0.00115^{1.5}$	$0.0025^{3}$	$0.0023^{1.5}$	$0.0023^{1.5}$
		$\hat{\lambda }$	$0.00281^{3}$	$0.00269^{1.5}$	$0.00269^{1.5}$	$0.00267^{3}$	$0.00242^{1.5}$	$0.00242^{1.5}$
		$\hat{\beta }$	$0.00486^{3}$	$0.0042^{1}$	$0.00421^{2}$	$0.00056^{3}$	$0.00055^{1.5}$	$0.00055^{1.5}$
	MREs	$\hat{\alpha }$	$0.05782^{1}$	$0.05785^{3}$	$0.05784^{2}$	$0.05264^{1}$	$0.05666^{2}$	$0.05672^{3}$
		$\hat{\lambda }$	$0.02928^{1}$	$0.03206^{2.5}$	$0.03206^{2.5}$	$0.028^{1}$	$0.02872^{2.5}$	$0.02872^{2.5}$
		$\hat{\beta }$	$0.02975^{1}$	$0.03449^{2}$	$0.03452^{3}$	$0.07612^{1}$	$0.07816^{3}$	$0.0781^{2}$
	∑Ranks		$19^{3}$	$16.5^{1}$	$18.5^{2}$	$21^{3}$	$16.5^{1.5}$	$16.5^{1.5}$
200	BIAS	$\hat{\alpha }$	$0.02154^{1}$	$0.02155^{2.5}$	$0.02155^{2.5}$	$0.03753^{1}$	$0.03761^{2.5}$	$0.03761^{2.5}$
		$\hat{\lambda }$	$0.04265^{1}$	$0.04286^{2.5}$	$0.04286^{2.5}$	$0.04256^{1}$	$0.0427^{2.5}$	$0.0427^{2.5}$
		$\hat{\beta }$	$0.04503^{3}$	$0.0439^{1}$	$0.04391^{2}$	$0.01486^{1}$	$0.0149^{2.5}$	$0.0149^{2.5}$
	MSEs	$\hat{\alpha }$	$0.00069^{3}$	$0.00068^{1.5}$	$0.00068^{1.5}$	$0.00174^{3}$	$0.00172^{1.5}$	$0.00172^{1.5}$
		$\hat{\lambda }$	$0.00217^{3}$	$0.00212^{1.5}$	$0.00212^{1.5}$	$0.00218^{3}$	$0.00213^{1.5}$	$0.00213^{1.5}$
		$\hat{\beta }$	$0.00276^{3}$	$0.00255^{1.5}$	$0.00255^{1.5}$	$0.00034^{2}$	$0.00034^{2}$	$0.00034^{2}$
	MREs	$\hat{\alpha }$	$0.04167^{1}$	$0.04309^{2.5}$	$0.04309^{2.5}$	$0.04721^{1}$	$0.05015^{2.5}$	$0.05015^{2.5}$
		$\hat{\lambda }$	$0.02792^{1}$	$0.02857^{2.5}$	$0.02857^{2.5}$	$0.02769^{1}$	$0.02847^{2.5}$	$0.02847^{2.5}$
		$\hat{\beta }$	$0.02942^{3}$	$0.02926^{1}$	$0.02927^{2}$	$0.05764^{1}$	$0.05962^{2.5}$	$0.05962^{2.5}$
	∑Ranks		$19^{3}$	$16.5^{1}$	$18.5^{2}$	$14^{1}$	$20^{2.5}$	$20^{2.5}$
350	BIAS	$\hat{\alpha }$	$0.0203^{1}$	$0.02037^{2.5}$	$0.02037^{2.5}$	$0.03526^{1}$	$0.03547^{2.5}$	$0.03547^{2.5}$
		$\hat{\lambda }$	$0.03934^{1}$	$0.03967^{2.5}$	$0.03967^{2.5}$	$0.03949^{1}$	$0.03974^{2.5}$	$0.03974^{2.5}$
		$\hat{\beta }$	$0.04524^{1}$	$0.04536^{2.5}$	$0.04536^{2.5}$	$0.01203^{1}$	$0.01207^{2.5}$	$0.01207^{2.5}$
	MSEs	$\hat{\alpha }$	$0.00062^{2}$	$0.00062^{2}$	$0.00062^{2}$	$0.00158^{3}$	$0.00157^{1.5}$	$0.00157^{1.5}$
		$\hat{\lambda }$	$0.00187^{3}$	$0.00186^{1.5}$	$0.00186^{1.5}$	$0.00191^{3}$	$0.00188^{1.5}$	$0.00188^{1.5}$
		$\hat{\beta }$	$0.00251^{3}$	$0.00243^{1.5}$	$0.00243^{1.5}$	$0.00021^{2}$	$0.00021^{2}$	$0.00021^{2}$
	MREs	$\hat{\alpha }$	$0.03947^{1}$	$0.04075^{2.5}$	$0.04075^{2.5}$	$0.04727^{1}$	$0.0473^{2.5}$	$0.0473^{2.5}$
		$\hat{\lambda }$	$0.02616^{1}$	$0.02645^{2.5}$	$0.02645^{2.5}$	$0.02523^{1}$	$0.02649^{2.5}$	$0.02649^{2.5}$
		$\hat{\beta }$	$0.02953^{1}$	$0.03024^{2.5}$	$0.03024^{2.5}$	$0.04659^{1}$	$0.0483^{3}$	$0.04829^{2}$
	∑Ranks		$14^{1}$	$20^{2.5}$	$20^{2.5}$	$14^{1}$	$20.5^{3}$	$19.5^{2}$

**Table 10:** Partial and overall ranks of all estimation methods for the PMKE distribution.

Parameter	*n*	BSE	BLN	BGE
α=0.5,λ=0.25,β=0.75	20	3	2	1
	50	3	2	1
	100	3	2	1
	200	3	2	1
	350	3	2	1
α=1.5,λ=0.75,β=0.5	20	2	1	3
	50	2	3	1
	100	1	2.5	2.5
	200	2	2	2
	350	2	2	2
α=0.5,λ=1.5,β=1.5	20	2.5	1	2.5
	50	3	1	2
	100	3	1	2
	200	3	1	2
	350	1	2.5	2.5
α=0.75,λ=1.5,β=0.25	20	3	1	2
	50	3	1	2
	100	3	1.5	1.5
	200	1	2.5	2.5
	350	1	3	2
∑ Ranks		47.5	36	36.5
Overall Rank		3	1.0	2.0

**Table 11:** Discrimination measures of the PNKE model and other competing models.

Model	$-\hat{\ell }$	AIK	CAKI	BAI	HAQUI	ANDA	CRVMI	KOSM	*p*-Value	Est. Parameters
PMKE	77.7073	161.415	162.081	166.481	163.246	0.100126	0.0132035	0.0529824	0.999873	$\hat{\alpha }=0.273225\;\left(0.121243\right)$
										$\hat{\lambda }=7.55291\times 10^{-10}\;\left(6.62807\times 10^{-9}\right)$
										$\hat{\beta }=10.3448\;\left(4.21646\right)$
MKE	81.2693	166.539	166.863	169.916	167.76	0.499672	0.0635224	0.102386	0.795807	$\hat{\alpha }=2.92823\;\left(0.397681\right)$
										$\hat{\lambda }=0.0993581\;\left(0.00401557\right)$
APE	88.992	181.984	182.308	185.362	183.205	1.4759	0.224938	0.151628	0.316583	$\hat{\alpha }=211389\;\left(712556\right)$
										$\hat{a}=0.479077\;\left(0.0539462\right)$
APExE	86.9505	179.901	180.568	184.968	181.733	1.07046	0.137637	0.130774	0.500765	$\hat{\alpha }=35.6617\;\left(54.329\right)$
										$\hat{a}=0.579806\;\left(0.0712686\right)$
										$\hat{c}=7.13943\;\left(3.70158\right)$
BE	87.4599	180.92	181.586	185.986	182.752	1.35976	0.207706	0.127833	0.530394	$\hat{\lambda }=0.0576468\;\left(0.00181934\right)$
										$\hat{a}=7.75266\;\left(2.67815\right)$
										$\hat{b}=18.349\;\left(1.70836\right)$
E	113.319	228.639	228.744	230.327	229.249	8.60052	1.73703	0.363108	0.0000525	$\hat{\lambda }=0.159936\;\left(0.0252881\right)$
ExE	90.1427	184.285	184.61	187.663	185.507	1.74781	0.280048	0.154196	0.297513	$\hat{\alpha }=9.51462\;\left(33.3448\right)$
										$\hat{\lambda }=0.449842\;\left(0.0025389\right)$
GExE	87.8425	181.685	182.352	186.752	183.517	1.42203	0.218748	0.131057	0.497955	$\hat{\lambda }=0.10895\;\left(0.115928\right)$
										$\hat{\alpha }=10.5505\;\left(3.66103\right)$
										$\hat{\delta }=8.11926\;\left(10.1673\right)$
GLLE	86.6504	177.301	177.625	180.679	178.522	0.95874	0.0994804	0.12411	0.568832	$\hat{\alpha }=3.63222\;\left(0.495438\right)$
										$\hat{\lambda }=0.110877\;\left(0.00598631\right)$
HETE	80.4739	166.948	167.614	172.014	168.78	0.309664	0.0418137	0.0890764	0.908766	$\hat{k}=0.133305\;\left(0.129544\right)$
										$\hat{\lambda }=5.07541\;\left(4.74335\right)$
										$\hat{\alpha }=857.376\;\left(973.017\right)$
IPLE	89.1039	184.208	184.874	189.274	186.04	1.25729	0.132512	0.149449	0.33342	$\hat{\alpha }=22.5789\;\left(17.1289\right)$
										$\hat{\beta }=0.150774\;\left(0.113026\right)$
										$\hat{\lambda }=0.912785\;\left(0.188307\right)$
KE	83.1772	172.354	173.021	177.421	174.186	0.729348	0.0934665	0.109691	0.721617	$\hat{\beta }=4.26681\;\left(0.730358\right)$
										$\hat{\lambda }=374.878\;\left(516.677\right)$
										$\hat{\alpha }=0.04161\;\left(0.0213235\right)$
LNE	91.8827	187.765	188.09	191.143	188.987	3.49189	0.672297	0.226798	0.0326519	$\hat{\beta }=1.77494\times 10^{-28}\;\left(0.0702828\right)$
										$\hat{\theta }=0.0467047\;\left(0.0151681\right)$
LE	86.6504	177.301	177.625	180.679	178.522	0.95874	0.0994804	0.12411	0.568832	$\hat{\alpha }=3.63222\;\left(0.495438\right)$
										$\hat{\lambda }=0.110877\;\left(0.00598631\right)$
MOE	83.6026	171.205	171.529	174.583	172.426	0.548981	0.0589614	0.0912843	0.892799	$\hat{\alpha }=285.719\;\left(240.378\right)$
										$\hat{a}=0.883713\;\left(0.120305\right)$
MOLE										$\hat{\lambda }=0.965429\;\left(0.988828\right)$
										$\hat{\theta }=290.349\;\left(246.823\right)$
	88.6703	183.341	184.007	188.407	185.172	1.41112	0.207381	0.144869	0.370713	$\hat{\alpha }=8.54642\;\left(3.17075\right)$
TGE										$\hat{\lambda }=-0.650755\;\left(0.256039\right)$
										$\hat{\theta }=0.500041\;\left(0.0621707\right)$

## References

[1] Al-Babtain A.A., Shakhatreh M.K., Nassar M., Afify A.Z. (2020). A New Modified Kies Family: Properties, Estimation Under Complete and Type-II Censored Samples, and Engineering Applications. Mathematics.

[2] Ghitany M., Al-Mutairi D., Balakrishnan N., Al-Enezi L. (2013). Power Lindley distribution and associated inference. Comput. Stat. Data Anal..

[3] Krishnarani S. (2016). On a power transformation of half-logistic distribution. J. Probab. Stat..

[4] Rady E., Hassanein W., Elhaddad T. (2016). The power Lomax distribution with an application to bladder cancer data. SpringerPlus.

[5] Barco K., Mazucheli J., Janeiro V. (2017). The inverse power Lindley distribution. Commun. Stat.-Simul. Comput..

[6] Habibi M., Asgharzadeh A. (2018). Power binomial exponential distribution: Modeling, simulation and application. Commun. Stat.-Simul. Comput..

[7] Bakouch H., Khan M., Hussain T., Chesneau C. (2019). A power log-Dagum distribution: Estimation and applications. J. Appl. Stat..

[8] Al-Omari A., Alhyasat K., Ibrahim K., Abu Bakar M. (2019). Power length-biased Suja distribution: Properties and application. Electron. J. Appl. Stat. Anal..

[9] Sobhi A.L., Mashail M. (2020). The Inverse-Power Logistic-Exponential Distribution: Properties, Estimation Methods, and Application to Insurance Data. Mathematics.

[10] Afify A.Z., Aljohani H.M., Alghamdi A.S., Gemeay A.M., Sarg A.M. (2021). A New Two-Parameter Burr–Hatke Distribution: Properties and Bayesian and Non-Bayesian Inference with Applications. J. Math..

[11] Nadarajah S., Kotz S. (2006). The beta exponential distribution. Reliab. Eng. Syst. Saf..

[12] Mansoor M., Tahir M., Cordeiro G.M., Provost S.B., Alzaatreh A. (2019). The Marshall-Olkin logistic-exponential distribution. Commun. Stat.-Theory Methods.

[13] Gupta R., Kundu D. (2001). Exponentiated exponential family: An alternative to gamma and Weibull distributions. Biom. J. J. Math. Methods Biosci..

[14] Pinho L.G.B., Cordeiro G.M., Nobre J.S. (2015). The Harris extended exponential distribution. Commun. Stat.-Theory Methods.

[15] Marshall A.W., Olkin I. (1997). A new method for adding a parameter to a family of distributions with application to the exponential and Weibull families. Biometrika.

[16] Artzner P. (1999). Application of coherent risk measures to capital requirements in insurance. N. Am. Actuar. J..

[17] Ristić M.M., Balakrishnan N. (2012). The gamma-exponentiated exponential distribution. J. Stat. Comput. Simul..

[18] Tian Y., Tian M., Zhu Q. (2014). Transmuted linear exponential distribution: A new generalization of the linear exponential distribution. Commun. Stat.-Simul. Comput..

[19] Lan Y., Leemis L.M. (2008). The logistic–exponential survival distribution. Nav. Res. Logist. (NRL).

[20] Nadarajah S., Haghighi F. (2011). An extension of the exponential distribution. Statistics.

[21] Khan M.S., King R., Hudson I.L. (2017). Transmuted generalized exponential distribution: A generalization of the exponential distribution with applications to survival data. Commun. Stat.-Simul. Comput..

